# Breast Milk, a Source of Beneficial Microbes and Associated Benefits for Infant Health

**DOI:** 10.3390/nu12041039

**Published:** 2020-04-09

**Authors:** Katríona E. Lyons, C. Anthony Ryan, Eugene M. Dempsey, R. Paul Ross, Catherine Stanton

**Affiliations:** 1 Teagasc Food Research Centre, Moorepark, Fermoy, Co. Cork P61 C996, Ireland; 2 School of Microbiology, University College Cork, Cork T12 YN60, Ireland; 3 APC Microbiome Ireland, University College Cork, Cork T12 YT20, Ireland; 4 Department of Neonatology, Cork University Maternity Hospital, Cork T12 YE02, Ireland; 5 INFANT Research Centre, University College Cork, Cork T12 DFK4, Ireland

**Keywords:** mammary gland, breast milk, human milk oligosaccharides (HMOs), human milk microbiome, lactation, probiotic, entero-mammary pathway

## Abstract

Human breast milk is considered the optimum feeding regime for newborn infants due to its ability to provide complete nutrition and many bioactive health factors. Breast feeding is associated with improved infant health and immune development, less incidences of gastrointestinal disease and lower mortality rates than formula fed infants. As well as providing fundamental nutrients to the growing infant, breast milk is a source of commensal bacteria which further enhance infant health by preventing pathogen adhesion and promoting gut colonisation of beneficial microbes. While breast milk was initially considered a sterile fluid and microbes isolated were considered contaminants, it is now widely accepted that breast milk is home to its own unique microbiome. The origins of bacteria in breast milk have been subject to much debate, however, the possibility of an entero-mammary pathway allowing for transfer of microbes from maternal gut to the mammary gland is one potential pathway. Human milk derived strains can be regarded as potential probiotics; therefore, many studies have focused on isolating strains from milk for subsequent use in infant health and nutrition markets. This review aims to discuss mammary gland development in preparation for lactation as well as explore the microbial composition and origins of the human milk microbiota with a focus on probiotic development.

## 1. Introduction

Human breast milk provides critical nutrients and bioactive compounds which support growth and immune development during infancy. Variation in milk constituents and bioactive compounds as a result of demographic and genetic factors, maternal lifestyle, and exposures can have both positive and negative effects on infant health [1,2,3]. In order for breast feeding to occur, the mammary gland must undergo a series of developmental changes which begin in utero and continue until birth. During this time frame, the mammary gland branches and elongates forming a fully functioning milk secreting network which is activated following delivery. Breastfed infants are reported to have a dynamic gut microbiome and have reduced incidences of certain diseases [4,5]. As a result, human breast milk has been largely investigated to unravel its unique composition, with infant formula manufacturers aiming to mimic breast milk composition for infants who are formula-fed. Advances in breast milk research has led to advances in infant formula science, resulting in the addition of bioactive compounds such as human milk oligosaccharides (HMOs) which have a bifidogenic effect, lactoferrin which plays a role in gastrointestinal and brain development, and choline which is also important for infant brain development [6]. Although human milk remains the gold standard feeding regime, demand for infant milk formula will continue as breastfeeding is not feasible in every case.

In addition to macro and micronutrients and bioactive compounds, human breast milk contains a plethora of bacterial species. In the past, bacteria isolated from breast milk were considered a contaminant from mother’s skin and infant oral cavity or from incorrect handling or storage methods [7,8]. However, it is now widely accepted that breast milk has its own unique microbiome, consisting of many commensal bacteria. Breast milk plays a vital role in inoculating the infant gut with bacteria after birth. Acknowledgement of the human milk microbiome as an asset to infant health has resulted in numerous investigations to elucidate its mechanisms of action, which include production of antimicrobial compounds, preventing adhesion of pathogenic bacteria to intestinal epithelium and enhancing intestinal mucin production [9,10,11]. Furthermore, the origin of bacteria in breast milk has led to much debate, with multiple studies investigating potential pathways that give rise to bacteria in milk such as retrograde backflow and the possible existence of an entero-mammary pathway [12].

As the health promoting benefits of bacteria in breast milk have been established, the isolation of potential probiotic strains from milk has been the focus of some investigations [9,11]. The isolation of potential probiotic strains has been limited to traditional bacterial species of *Bifidobacterium* and *Lactobacillus* due to a long track record of safety and efficacy in nutrition and health markets. However, research looking at unconventional bacterial species may shed new light on these as possible probiotics to improve overall gut health. This review provides an overview of mammary gland development in preparation for breastfeeding and lactation, milk nutrient composition, bioactive and microbial composition, and relationship between human milk and infant health and development. This review particularly focuses on milk microbial composition and probiotic potential of milk-derived strains to enhance infant gut and immune development as well as potential in the health market.

## 2. Mammary Gland Development

### 2.1. Mammary Gland Development In Utero

The human breast begins to develop in utero, as early as four to six weeks gestation [13]. During this timeframe, paired thickenings known as mammary ridges or milk lines develop on the abdominal surface of the embryo. By week 7, the milk lines shorten and thicken into small nodules comprised of ectodermal cells [14]. Towards the end of the first trimester, these nodules descend into the embryonic connective tissue to form a mammary bud which is regulated by mesenchymal interactions and secretions [15,16].

In the second trimester, the mammary bud begins to enlarge and branch, yielding secondary epithelial buds which grow downwards into the mesenchyme. These buds continue to grow, branch and elongate, and coalesce to form lactiferous ducts. The branching morphogenesis of the secondary bud requires soluble factors for the production of hormones and growth factors, which promote and regulate growth of the mammary gland [17,18]. At the end of the second trimester, the basic structure of the mammary gland is established.

Continued branching and canalisation of the mammary buds occurs throughout the third trimester. By the end of gestation, each mammary bud has developed 15–20 lobular structures each containing lactiferous ducts. The mesoderm surrounding the area of internal growth proliferates resulting in the formation of an inverted nipple. By the fifth month of gestation, the areola surrounding the nipple is formed, and the epidermis above the inward growth becomes depressed and forms the mammary pit. Meanwhile the lactiferous ducts canalise and drain into the retroareolar ampullae which converge to open at the tip of the nipple [16,19,20]. At birth, the developing breast and mammary gland consists of a functioning network of mammary lobes and branching lactiferous ducts surrounded by connective tissue [21].

As maternal hormone influences subside, it has been reported that the newborn mammary gland undergoes stimulation at early infancy through a surge of the infant’s own reproductive hormones. Schmidt et al. reported that infant females aged 2–4 months had significantly higher estradiol levels than infant males, and this was positively correlated with breast tissue size [22]. Furthermore, higher estradiol levels in infant girls results in breast tissue persisting for longer when compared to infant males [21,22,23].

After birth, the inverted nipple becomes evert, and the areola darkens in pigmentation [18]. Anbazhagan et al. documented the morphological and functional changes in the breast from birth to two years of age, detailing three stages of morphological change outlining the branching ductal system and four stages of functional changes discussing the secretory capacity of the lining epithelium [24]. By two years of age, mammary gland development remains relatively inactive until puberty [25,26].

### 2.2. Mammary Gland Development During Puberty and Pregnancy

Pubertal changes in the breast and mammary gland are largely due to the influence of growth and sex hormones. Driven by the influence of estrogen, proliferation of epithelial cells results in an increase in fibrous and fatty tissue in the breast. The epithelium develops a network of branching bundles of ducts which form terminal end buds. Branching and ductal elongation occurs at the site of the terminal end bud resulting in the formation of alveolar buds and several terminal ductules. Each alveolus is enclosed in a bundle of contractile myo-epithelial cells. This complex structure, composed of the terminal duct and a collection of terminal ductules or acini, is known as terminal duct lobular unit (TDLU), which is the structural unit of the adult breast [14,27,28]. With each menstrual cycle, more alveoli are laid and ductal elongation and side branching continues due to circulating estrogen and progesterone [29]. Adipose tissue as well as blood vessels, fibroblasts and immune cells occupy the remaining area in the breast [30]. However, maximum maturation and development of the alveolar cells for milk secretion only occurs during pregnancy under the influence of hormones.

Around the twelfth week of pregnancy, lobules increase rapidly in number as a result of cell division and increased epithelial surface area. By mid-pregnancy, the enlarged lobules surround the central branching duct and the terminal duct can no longer be recognized. Further cell proliferation and differentiation results in milk producing cells, and the gland progresses into the secretory initiation phase [31,32]. The formation of secretory acini and the differentiated structures become progressively noticeable during this time. Although mammogenesis begins during puberty, it is not fully completed until pregnancy. With regard to lobular formation, there are four distinct lobular structures in the human breast. Lobule 1 is composed of a short terminal ductile, which progresses to lobule 2 and 3 due to proliferation, ductal elongation, and branching. In turn, lobule 3 progresses to lobule 4 in women who have given birth and completed lactation.

## 3. Lactogenesis

Lactation is defined as the secretion of milk from the mammary gland and is influenced by a complex hormonal network. Lactogenesis occurs in two distinct phases: lactogenesis 1 and lactogenesis 2. Lactogenesis 1, also known as initiation of lactation occurs mid-pregnancy and is defined by the secretory differentiation of the alveolar mammary epithelial cells into lactocytes which have the capacity to synthesize milk components [33,34]. During this time, the gland is sufficient to secrete small quantities of protein rich fluid which is expelled into the mammary alveoli and discharged from the nipple. This secretion is referred to as colostrum; however, high levels of progesterone typically inhibit milk secretion before birth [35,36,37].

After birth, the expulsion of the placenta results in an abrupt decrease in progesterone and estrogen, coupled with an increase of prolactin, insulin, and cortisol thus stimulating copious milk production and therefore onset of lactogenesis stage 2 [38,39]. During this time, the ability of the mammary epithelial cells to synthesise milk rapidly develops, and milk volumes were reported to increase from ~100 mL to 500 mL by four days postpartum, and 650 mL by eight days postpartum, and it is hormonally driven by the endocrine system [40,41]. Lactogenesis stage 2 is reported as “delayed” if the onset of copious milk production has not occurred by 72 h postpartum [33,42].

Milk production is dependent on a “supply demand” process, and milk removal is the primary control mechanism for maintaining supply. Oxytocin is essential for milk removal from the mammary gland [43]. Infant suckling triggers the release of oxytocin from the posterior pituitary which interacts with myo-epethelial cell receptors located on the differentiated alveoli and lactiferous ducts. This results in the contraction of cells enabling the secretion of milk from the mammary gland. Milk synthesis is under the control of a polypeptide called “feedback inhibitor of lactation”, which regulates milk production once lactation has been established [44]. If breast milk is not removed by infant sucking or expression, feedback inhibitor of lactation builds up leading to a decrease in milk production and ultimately mammary involution. Cessation of breast feeding results in decreased milk production by apoptosis of milk synthesising epithelial cells [38,45]. During mammary involution, the mammary gland undergoes extensive tissue remodelling and reverts back to a non-pregnant state.

## 4. Milk Synthesis

In order for milk synthesis to occur, prolactin must be present. Although initially required for the morphological development and differentiation of the mammary gland, prolactin plays a crucial role in stimulating milk protein and lactose synthesis [46,47]. Necessary nutrients and elements needed for milk synthesis reach the mammary epithelial cells in the mammary gland through the blood and lymph system where they are secreted into milk by several highly regulated transport routes known as the paracellular and transcellular pathways. This includes one paracellular pathway, which involves exchange of substances passing through the intercellular space between the cells, and four transcellular pathways, which allows transport through the cell, passing through both apical and basolateral membranes. Entry of molecules via paracellular and transcellular pathways is regulated by hormones and growth factors [37,48].

Endogenously produced substances such as major milk proteins, lactose, oligosaccharides, citrate, calcium, and phosphate are secreted through the exocytic pathway. These substances are enveloped into secretory vesicles within the golgi, and are transported to the apex of the cell membrane where they merge with the plasma membrane excreting their contents into the extracellular space. Lipids and lipid-like proteins are synthesised within the cytoplasm of the mammary alveolar cells and are secreted in a budding process unique to mammary epithelial cells. Triglycerides and phospholipids synthesised in the mammary alveolar cells from precursor fatty acids and glycerol combine to form large droplets. These lipid droplets become enclosed in the apical plasma membrane and are secreted as membrane enveloped structures called milk fat globules [49].

Pathways involving membrane transport allow for the transfer of ions, glucose, amino acids, and trace elements from blood to milk in the mammary gland and rely on a number of factors such as the combined activity of the apical and basal plasma membranes and various transport proteins. Ion transporters for potassium, chloride and sodium have been identified on both the apical and basal plasma membranes of mammary alveolar cells, whereas transporters for calcium, iodine, citrate, and phosphate appear to be limited to the basal plasma membrane. Sodium and potassium are also transported via Na+/K+ ATPase pumps located in the basal plasma membrane of the mammary epithelial cells. Adequate supply of trace elements is crucial to ensure neonatal survival and optimum health [50,51,52,53].

Glucose is a substrate required for important metabolic processes in the mammary epithelial cells such as the synthesis of lactose and is transported via two specific glucose transport mechanisms: glut1 and sodium dependent glucose transporter. These transport pathways are found on the apical and basal membranes as well as the golgi and secretory membranes [54]. To date, both sodium-dependent and sodium independent amino acid transport mechanisms have been identified at the basal membrane of the mammary epithelium; however, it remains uncertain if similar amino acid transport systems are present on the apical membrane [55].

The transcytic pathway allows for the transport of several macromolecules derived from serum or stromal cells. Proteins such as immunoglobulins, transferrin and albumin, hormones such as prolactin, oestrogen, and insulin, and secretory antibodies, cytokines, and lipoprotein lipase undergo vesicular transcytosis from the interstitial space. These pathways involve endocytic uptake of the molecule which is subsequently transported across the cell and secreted by exocytosis [51,56]. In summary, the mandatory components needed for milk synthesis reach the mammary gland through several highly regulated transport mechanisms.

## 5. Milk Composition

**Figure 1:**
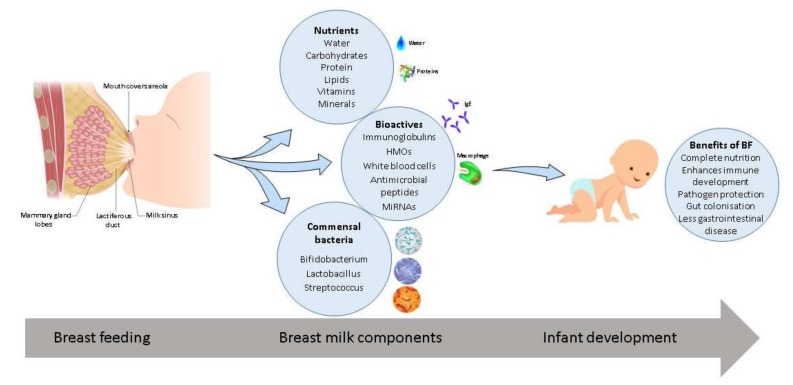
Breast milk composition and associated benefits. Breast milk provides essential nutrients, bioactive compounds, and commensal bacteria which aid in growth and development of the infant and the immune system. Associated benefits of breast feeding (BF) include protection against pathogens, enhanced immune development, complete nutrition, promotion of gut colonization, and less incidences of gastrointestinal disease.

Over the last few years, there has been an increasing appreciation and emphasis on promoting breast milk feeding for enhancing infant health, growth, and development. Human breast milk is the gold standard feeding regime for newborn infants. It is composed of the correct amount of nutrients and bioactive compounds to provide complete nutrition for the developing infant as well as beneficial bacteria which protect vulnerable immune systems against disease (Figure 1). Breast milk is essentially a dynamic biological fluid which changes in composition over the course of lactation to meet the needs of the growing infant [57]. Milk composition varies between mothers who have given birth full-term and preterm. The World Health Organization recommends mothers worldwide to breastfeed infants for the first six months of life to achieve optimal growth, development, and health [58].

### 5.1. Macro and Micronutrient Composition

Human milk changes in composition from colostrum to transitional milk to mature milk over the course of lactation. Colostrum, the first fluid produced by mothers after parturition, occurs in small quantities during the first two to four days [59]. It is distinct from mature milk in terms of colour, composition, and consistency. Although the nutrients in colostrum and mature milk remain similar, levels of the nutrients vary throughout lactation. Colostrum is rich in whey proteins and minerals, however, it contains lower levels of lactose and fats and certain vitamins when compared to mature milk. While having higher levels of chloride, sodium, and magnesium, it has lower levels of calcium and potassium when compared to mature milk [60,61,62].

Transitional milk represents a period of increased milk production occurring from five days to two weeks postpartum and is similar to the characteristics of colostrum. This “ramped up” production of milk is to support the growth and nutritional needs of the developing infant. From two weeks postpartum, human milk is considered fully mature milk [63]. While fluctuations in milk composition levels are observed over the first month of life, human milk remains relatively akin in composition, although slight changes in milk nutrient concentrations do occur throughout the course of lactation. Mature milk reportedly contains 3–5% fat, 6.9–7.2% carbohydrate calculated as lactose, 0.8–0.9% protein, and 0.2% mineral constituents [64].

The most abundant proteins in human milk are casein, lactoferrin, α-lactalbumin, lysozyme, secretory immunoglobulin IgA, and serum albumin [65,66]. High protein concentrations are apparent in colostrum and milk during the first few weeks, however, a steady decrease is observed thereafter [67,68]. Lipids form an important part of human milk and are the main source of energy. Fat content in milk varies throughout feeding, with higher concentrations of milk fat found in hind milk when compared to foremilk [67]. It is reported that fat composition in milk is influenced by a number of factors such as diet and parity of the mother [69,70]. Lactose is the major carbohydrate in milk and while it is relatively low in colostrum, it rapidly increases and remains constant throughout the course of lactation [67,71].

Although many advances have been made in infant formula manufacturing and addition of multifunctional bioactives to improve infant health is being investigated, it lacks the ability to vary throughout daily feeding and evolve over time to match the needs of the developing infant. A detailed review documenting the advances in infant formula was outlined by Ahern et al. [6].

### 5.2. Bioactives

In addition to providing correct nutrients required for energy, human milk provides many bioactive components and immune factors such as antibodies, immunoglobulins, lactoferrin, lysozyme, antimicrobial peptides, growth factors, white blood cells, microRNAs, and human milk oligosaccharides (HMOs) which play a vital role in boosting the developing infant immune system and providing defence against pathogens [72]. It has been reported that colostrum contains higher levels of immunoglobulins, cytokines, and immune cells when compared to mature milk [73,74,75]. Bioactive compounds in milk come from numerous sources, many are produced and secreted by the mammary epithelium and cells in milk while others are transferred across the mammary epithelium by receptor-mediated transport from maternal serum [76,77]. For the purpose of this review, we focus on a select number of bioactive compounds which vary across the course of lactation.

#### 5.2.1. Human Milk Oligosaccharides

Human milk oligosaccharides (HMOs) are complex glycans present in high quantities in breast milk, 20–25 ng/L in colostrum, and gradually declining to 5–15 ng/L in mature milk [78]. Over 200 HMOs to date have been identified, and they vary in structure and composition in milk over the course of lactation [79]. HMOs, although indigestible to the infant, are the third most abundant component of human milk, after lactose and lipids, which function to nourish bacterial communities in the infant gastrointestinal tract (GIT) [80]. Essentially, HMOs are considered as prebiotic agents that serve as metabolic substrates which give rise and promote the growth of beneficial microorganisms in the infant intestinal microbiome. As well as promoting the growth of commensal bacteria, HMOs have been reported to modulate intestinal epithelial cell responses and pathogen deflection and prevent pathogen adhesion to intestinal epithelium. HMOs prevent the attachment of pathogenic bacteria by serving as soluble glycan receptor decoys. It has been reported that HMOs resemble structures of viral receptors and prevent adherence to cells, therefore preventing infection [81,82,83]. A number of studies have documented that HMOs play an important role in preventing infant gastrointestinal and respiratory tract infections [84,85].

Studies have shown that beneficial health enhancing microbes such as *Bifidobacterium* spp. are adapted for utilisation of HMOs in the infant gut [86,87], while limiting the growth of potentially harmful bacteria [88,89]. Many investigations have reported that breast-fed infants have a higher abundance of beneficial *Bifidobacterium* spp. compared with formula-fed infants [90,91]. As HMOs are absent from infant formula, attempts to mimic their multiple benefits has resulted in the addition of other non HMO prebiotics such as fructooligosaccharide (FOS) and galactooligosaccharide (GOS) with the aim to stimulate beneficial bacterial growth in formula fed infants [92,93]. Several studies suggest that supplementation with FOS and GOS encourages a *Bifidobacterium* spp. dominated infant and adult gut microbiome [94,95].

Utilisation of HMOs has only been identified in certain *Bifidobacterium*, *Lactobacillus*, and *Bacteroides* spp., and it has been reported that different strains utilise different enzymatic mechanisms and protein-substrate binding to metabolise HMOs [87,89,96,97]. *Bifidobacterium longum* ssp. *infantis* commonly found in the gut of breast-fed infants, utilises HMOs which enhances gut colonization [98]. It has been reported that *B. longum* ssp. *infantis* possesses the ability to utilise several types of HMOs, whereas different strains of *Bifidobacterium bifidum* are capable of using fucosylated or sialylated HMOs [99,100].

In vitro studies have documented the growth of *B. longum* ssp. *infantis* on HMOs which increased the expression of anti-inflammatory cytokine interleukin-10 as well as increasing adhesion to epithelial cells [101,102]. A subsequent study by the same group determined that *B. longum* ssp. *infantis* when grown on HMOs resulted in a higher percentage binding to Caco-2 cell monolayers when compared to lactose and glucose grown *B. longum* ssp. *infantis* [102]. Lawson et al. reported that different *Bifidobacterium* strains from the same infant have overlapping but distinct HMO utilisation abilities [103]. Mechanisms of HMO utilisation of *Bifidobacterium breve, Bifidobacterium bifidum and Bifidobacterium adolescentis* have also been investigated [104,105,106,107].

The safety of synthetic HMOs as 2-fucosyllactose (2FL) has been assessed in vitro and in animal trials with no adverse health effect reported [108]. Following this, Goehring et al. looked at the immune effects of infant formula supplemented with HMOs 2FL in healthy full-term infants. Results demonstrated that infants fed formula supplemented with 2FL had statistically similar levels of five immune markers when compared to breast fed infants. Infants fed supplemented 2FL formula displayed lower inflammatory cytokine profiles, similar to that of breast fed infants, in comparison to non-supplemented formula fed infants [109].

More recently, it has been documented that some HMOs exhibit antimicrobial and antibiofilm properties against group *B Streptococcus* (GBS). GBS is a major cause of infection of the fetal membranes (chorioamnionitis), which can lead to neonatal sepsis, preterm birth, and still birth. HMOs also showed antibacterial activity against *Acinetobacter buamannii* which can cause blood and urinary tract infections as well as antibiofilm activity against methicillin-resistant *Staphylococcus aureus* [110,111,112].

#### 5.2.2. MicroRNA

MicroRNAs or miRNAs are small noncoding RNA molecules that play an important role in the regulation of gene expression at the post-transcriptional level [113]. MicroRNAs have been identified across plants, animals, and viruses and within a number of human body fluids such as blood, breast milk, urine, and saliva [114,115,116,117,118,119,120,121]. These molecules can be secreted into extracellular fluid where they can be packaged into exosomes, which are membrane vesicles which contain various microRNAs [122,123,124]. Extracellular microRNAs are extremely stable and capable of withstanding low pH [121]. Among different body fluids, human milk is one of the most abundant sources of microRNAs [125], and, along with other bioactive molecules present in milk, they are suggested to play a role in infant development. Within breast milk, microRNAs appear to originate from the mammary gland, while the maternal circulation plays a smaller role [126]. Their isolation has been demonstrated from various fractions of milk including lipid, cell, and skim milk fractions, with the lipid and cell fractions being noted to contain a larger proportion of microRNAs when compared to skim milk [127]. The microRNA profile of human milk has also been compared between mothers of full-term versus mothers of preterm infants. A study by Carney et al. identified differences in the expression profiles of nine miRNAs in the skim milk and lipid fractions of preterm breast milk when compared to full-term samples [128]. Furthermore, a recent investigation by Shiff et al. noted that miRNA-320 was expressed less in preterm milk while miRNA-148 was more expressed when compared to full-term milk [129]. It has also been shown that human breast milk contains a higher proportion of microRNAs when compared to infant formula [130].

The function of microRNAs in breast milk has been the subject of much investigation, with proposed mechanisms relating to immune and metabolic functions. One such role may be to aid in the development of the infant’s immune system. During the first six months of lactation, Kosaka et al. observed high expression levels of immune related miRNAs, such as miR-155 which is involved in regulation of the innate immune system and B- and T- cell maturation [131]. Zhou et al. also noted immune related miRNAs were rich in breast milk exosomes. As mentioned previously, the microRNA profile differs between full-term and preterm breast milk [128]. Carney et al. also noted that the gene targets of the nine microRNAs that differed between both groups influenced metabolic processes such as lipid metabolism. Additionally, in vitro studies using mammary epithelial cells suggest a role for mir-221, previously identified in milk, in the regulation of lipid metabolism [132].

The role of microRNAs in biological processes has been well documented; however, with regard to human breast milk, more work is needed to fully understand the function of microRNAs and their contribution to this environment and even more so their role in infant development.

#### 5.2.3. Other Bioactive Compounds

Lactoferrin, the second most abundant protein in human milk, is an iron binding glycoprotein involved in a variety of immune functions. It displays antimicrobial and anti-infectious activity, with many clinical trials reporting its preventative role in neonatal sepsis, diarrhea, and necrotizing enterocolitis in preterm infants. High levels of lactoferrin are reported in colostrum 7 ng/L, with a gradual decline to 2–4g/L in mature milk [133,134,135,136,137]. Human milk contains an array of specialised growth factors such as epidermal growth factor, which aids healing of intestinal mucosa, insulin-like growth factors (IGF) 1 and 2 which increase tissue growth, and neuronal growth factors which help peristalsis; these are just some of the growth factors present in human milk that enhance infant development [138,139,140,141]. Secretory IgA (SIgA) and SIgG are the most abundant immunoglobulins in milk and are present in high concentrations during early lactation. These antibodies provide much needed immune protection to the newborn infant [142]. It has been reported that SIgA, the primary protective antibody in breast milk, is present at concentrations up to 12 mg/mL in colostrum and prevents pathogen adherence to epithelial cell surfaces and neutralises toxins [143,144].

### 5.3. Preterm Milk Composition

Preterm births are associated with nutritionally compromised infants with underdeveloped, immature immune systems, and therefore are more at risk of necrotizing enterocolitis and long term health implications [145,146,147]. Human breast milk is recommended as the first feeding regime for preterm infants to enhance the growth and development of the hindered immune system to match that of full-term infants [148]. However, in situations where the neonate is very preterm or has a low birth weight, there is a risk of inadequate nutrients in mother’s milk, necessitating its fortification with nutrients to ensure sufficient energy, protein, and micronutrient intake [149,150].

Differences in macronutrient composition are apparent between preterm milk samples and full-term milk samples. Breast milk from women who have given birth prematurely varies in composition, with increased levels of protein, fat, and many bioactive molecules compared to milk from women who have given birth full-term [151]. Initially, preterm milk is significantly higher in protein, fats, sodium, and free amino acids; however, a decrease in these nutrients is observed over the first few weeks following delivery [67]. Although full-term and preterm milk have similar mineral and trace element content, calcium is present in significantly lower levels in preterm milk and does not increase over time. Copper and zinc are found in higher quantities in preterm milk compared to full-term milk [152,153]. Furthermore, higher levels of many bioactives and immune factors are documented in preterm milk, such as higher levels of epidermal growth factor, SIgA, and HMOs [154,155,156]. In addition, it has been reported that during the first few days of lactation, preterm milk contains higher levels of lysozyme and lactoferrin than full-term milk [157].

## 6. Infant Formula

When maternal breast milk is not an option, artificial infant milk formula must supply fundamental nutrients to the newborn. Many determinants influence the decision to breast feed or formula feed including family support, employment, medical issues, complications during pregnancy and labour, and supply issues [158,159]. In circumstances where breastfeeding is not feasible, infant formula becomes the staple diet for the newborn. Although there have been advances in infant formula manufacturing, the production of formula identical to breast milk is not feasible. While every effort has been made to produce an effective substitute mimicking breast milk, discrepancies in nutrients and other components are apparent. There are many different formula options available; these can be derived from cows-milk, goats-milk, be soy-based, and can also include specialised formulations, e.g., goodnight and hypoallergenic formulas which all meet the nutritional needs of the developing infant [160,161].

While infant formula is predominantly derived from bovine milk, differences exist in composition and quantities of fats, proteins, vitamins, and minerals when compared to human milk [162]. Furthermore, bovine milk contains higher levels of protein, fats, and minerals but lower levels of lactose, resulting in the need to undergo modifications to resemble that of human milk [163,164,165]. The total protein content of bovine milk ranges from 1.80 to 2.0 g/L, and it has been reported that during infancy, high protein consumption is linked with faster weight gain and obesity in later life [166]. Caseins are present in higher amounts in bovine milk than in human milk. However, it has been reported that bovine caseins may be more difficult to digest for the infant, and symptoms of allergy to bovine milk can occur within the first year of infancy [164,167].

Infant formula lacks the diverse bacterial communities present in human breast milk. The addition of prebiotics to formula is a common practice to promote the growth of commensal bacteria in the neonatal gut. Food-grade oligosaccharides which are approved for use in infant formulas include FOS, GOS, polydextrose (PDX), lactulose (LOS), and inulin [92]. Some studies have documented positive health outcomes associated with prebiotic-supplemented formula. An investigation by Arslanoglu et al. reported that infants who were given long chain FOS and short chain GOS supplemented formula had fewer upper respiratory tract infections, fewer infections requiring antibiotic treatment, and overall fewer incidences of reoccurring infection [168]. Furthermore, it has been reported that formula supplemented with FOS and GOS have resulted in significant reductions in infant asthma prevalence as well as a significant reduction in eczema [169,170].

While supplementation of infant formula with beneficial health promoting bacteria commonly found in breast milk should be considered as a mechanism to promote infant health and gut colonisation in formula-fed infants, there are difficulties in ensuring the added probiotic bacteria survive GIT transit while also exerting beneficial health effects to the infant. Furthermore, extensive research needs to be carried out on the potential risks, safety, and efficacy of the probiotic-supplemented formula to infant health.

## 7. Breast Milk Microbiome

While initially considered a sterile fluid, several investigations have since identified breast milk as an integral source of microbes for the developing infant [171]. To date, many studies have concluded that breast milk is home to an array of bacterial species, its own unique microbiome, including beneficial, commensal, and potentially probiotic bacteria. This discovery has led to increased interest in the human milk microbiome and transfer of health promoting bacteria to infants [172,173,174,175]. It has been predicted that breast fed infants consume up to 8 × 10^5^ bacteria every day, with breast milk being the second integral source of microbes to the infant after the birth canal in vaginally born infants [7,176].

Historically, the microbiome of human breast milk has been limited to investigating transfer of potential infectious bacteria, such as those involved in clinical cases of mastitis [177,178,179,180]. Breast milk transmission from mother to infant of Q-fever causing bacteria *Coxiella burnetii* and infantile pneumonia causing leukocidin-producing *S. aureus* have also been reported [181,182]. Other reports focused on the contamination of breast milk due to incorrect collection and storage and subsequent implications to newborns [183,184]. However, it has been concluded to be bacteriologically safe to refrigerate expressed breast milk for up to 48 h [185]. Previous reports also suggested the milk microbiome was as a result of contamination of bacteria from maternal skin and infant mouth [7,8,186].

Evidence of milk’s own microbiome first stemmed from culture-based investigations; however, this approach has many limitations with certain bacterial species being difficult to culture. Gavin and Ostovar isolated bacterial species belonging to five families: *Micrococcaceae, Streptococcaceae, Corynebacteriaceae, Lactobacillaceae*, and *Neisseriaceae* from breast milk of five lactating women [8]. Over time, a number of culture-based studies continued to identify and isolate bacterial species in breast milk belonging to *Staphylococcus* spp. (*S. aureus*, *S. epidermidis*), *Streptococcus* spp. (*S. salivarius*), *Enterococcus* spp., *Lactobacillus* spp., and *Bifidobacterium* spp. [7,171,187,188]. Among the genera isolated in milk, many species belonging to *Bifidobacterium* and *Lactobacillus* have been isolated, such as *Bifidobacterium breve, Bifidobacterium bifidum, Bifidobacterium adolescentis, Lactobacillus gasseri, Lactobacillus fermentum, Lactobacillus plantarum, Lactobacillus rhamnosus*, and *Lactobacillus salivarius* [9,11,189,190,191,192,193].

With advances in sequencing technologies, more detailed analysis of breast milk has enabled a better understanding of the microbiome composition and diversity, with over several hundred bacterial species identified [194]. Using a culture-independent approach, an investigation by Hunt et al. evaluated the microbiota of breast milk from 16 lactating women across three time-points over four weeks. Nine core genera were identified across milk samples including *Staphylococcus, Streptococcus, Serratia, Pseudomonas, Corynebacterium, Ralstonia, Propionibacterium, Sphingomonas*, and *Bradyrhizobiaceae* [195]. In comparison, Jost et al. investigated the microbiota of breast milk samples from seven lactating women across three different time-points. *Staphylococcus, Streptococcus, Bifidobacterium, Balutia, Brevundimonas, Corynebacterium, Flavobacterium, Propionibacterium, Pseudomonas, Ralstonia, Rothia*, and *Burkholderia* comprised the 12 most abundant genera [187]. Furthermore, Murphy et al. examined the microbial composition of breast milk and infant stool in 10 mother–infant pairs from birth to three months. This milk microbiota consisted of 12 core genera: *Pseudomonas, Staphylococcus, Streptococcus, Elizabethkingia, Variovorax, Bifidobacterium, Flavobacterium, Lactobacillus, Stenotrophomonas, Brevundimonas, Chryseobacterium*, and *Enterobacter* [174]. More recently, Chen et al. documented the microbiota in milk samples collected from 33 women with the five genera *Staphylococcus, Streptococcus, Enhydrobacter, Enterococcus*, and *Rothia* predominating [196]. While the focus of the investigations above was to determine the milk microbiome of healthy women following birth over lactation, differences in core genera among these studies is evident.

Variations in the milk microbiome may be attributed to many factors such as maternal diet, genetics, health, mode of delivery, demographic, or environmental differences [2,197,198,199]. A number of investigations have begun to assess the impact of these factors on the milk microbiome. Khodayar-Pardo et al. examined the impact of gestational age, mode of delivery, and lactation stage on the breast milk microbiota. In particular, this study determined that Cesarean section births were associated with higher overall total bacterial concentrations, in early lactation (days 1–16), with significantly higher levels of *Streptococcus* spp. and significantly lower levels of *Bifidobacterium* spp. when compared to vaginal deliveries [200]. Hermansson et al. also reported that the milk microbial composition was associated with significant changes as a result of birth mode and exposure to intrapartum antibiotics. However, despite differences in the microbiome of breast milk due to these factors, 18 bacterial families were shared between mothers [2]. The effects of antibiotherapy on the milk microbiota composition resulted in the detection of significantly lower *Bifidobacterium* and *Lactobacillus* spp. in samples from women who had received antibiotherapy during pregnancy and lactation [201]. It has also been noted that maternal body mass index (BMI) and weight gain during pregnancy have an impact on milk microbiome composition, with lower bacterial diversity observed in colostrum and one month milk samples from high BMI/obese women [199], while mothers with celiac disease have reduced levels of *Bifidobacterium* spp. in their milk [202]. With regards to demographics, Li et al. reported geographical differences in the milk microbiome profiles of women living in mainland China and Taiwan, while Kumar et al. also reported differences in the microbiota composition of breast milk in women from different geographical locations across Europe, Africa, and China [197,203]. Furthermore, alterations in sample collection, DNA extraction techniques and sequencing techniques can also contribute to variations in the milk microbiome [204,205]. Interestingly, despite differences between these studies, two genera, *Staphylococcus* and *Streptococcus* were constant core members of the milk microbiota.

Additionally, several investigations have observed that there is mother to infant vertical transfer of bacterial species [206,207,208,209,210]. Murphy et al. demonstrated vertical transfer through the isolation of viable *Bifidobacterium breve* and *Lactobacillus plantarum* from both mother’s milk and corresponding infant stool [174].

Microbiome analysis of breast milk from mothers of preterm infants is limited. This may be due to factors such as delayed onset of milk production or small volumes of milk which will be needed to aid the development of the preterm infants. Khodayar-Pardo et al. documented the milk microbiome following full-term (*n* = 13) and preterm birth (*n* = 19) over the first four weeks of lactation. *Bifidobacterium*, *Lactobacillus, Staphylococus, Streptococcus*, and *Enterococcus* were among the genera detected in both full-term and preterm milk samples. However, *Bifidobacterium* spp. were detected in significantly lower levels in the preterm group across all stages of lactation [200]. In contrast, an investigation by Urbaniak et al. reported no statistically significant differences in the bacterial profiles of breast milk samples following full and preterm birth [211]. Furthermore, Biagi et al. highlighted a change in the microbial composition of preterm milk following infant latching, with bacterial communities relating to the oral cavity being identified, in particular *Streptococcus* and *Rothia* [212]. In order to fully understand the benefits of breast milk composition for premature infants, more research is needed to determine the bacterial communities present in preterm milk over the course of lactation.

## 8. Origins of Milk Microbiome

Although it is now widely accepted that breast milk has its own microbiota, the origin of these bacterial populations in milk is not fully understood and has been subject to much debate. Traditionally, it was believed that the milk microbiome was as a result of contamination from mother’s skin during infant suckling, and many studies note the similarities between the adult skin microbiome and milk microbiome, particularly among the genus *Staphylococcus* and *Corynebacterium* [176,213]. Furthermore, bacteria from breast milk could be influenced by the infant oral cavity, where studies have reported, via ultrasound imaging, retrograde back flow of milk due to infant suckling. This back flow of milk into the mammary ducts provides one possible mechanism detailing the transfer of bacteria from infant’s mouth into the mother’s mammary gland [214,215,216]. Additionally, it was suggested that breast pump expression could influence the microbiome, proposing the pump could play a potential role in retrograde of exogenous bacteria into the milk ducts [217,218].

Moreover, it has been noted that there are changes in immunological composition of breast milk in response to active infant infection. Riskin et al. reported an increase in the number of white blood cells, in particular macrophages and TNFα levels, in breast milk during active infection in feeding infants [219]. Hassiotou et al. also documented an increase in breast milk leukocyte levels when the nursing infants had infections [220]. Although more research is needed, investigations suggest that during retrograde backflow, saliva from the infant’s oral cavity flows back into the mammary gland. This exposure of infant infection to mother may stimulate an immunomodulatory response, leading to increased leukocyte and antibody production in breast milk. These results further support the knowledge that breast milk changes in composition in response to infant infection and confers immunological protection to the infant.

However, the discovery of anaerobic species associated with gut environments that are unable to exist in aerobic environments has sparked interest into the complexity of the origins of bacteria in breast milk. These findings suggest that live bacteria from the maternal gut travel through an endogenous route to the mammary gland via the presence of an entero-mammary pathway. This translocation of bacteria from maternal gut to mammary gland involves complex interactions between epithelial cells, immune cells, and bacteria [12,221]. Evidence supporting the entero-mammary pathway includes the presence of bacterial communities in colostrum collected before first infant suckling [222].

The mechanism of physiologic translocation involves immune cells, dendritic cells, and CD18 cells which deliver nonpathogenic bacteria from the gut lumen to the lactating mammary gland. Dendritic cells are able to penetrate the gut epithelium by opening the tight junctions between intestinal epithelial cells and take up bacteria from the gut lumen [223]. Bacteria are subsequently transported by macrophages to mesenteric lymph nodes and ultimately to the mammary gland [12,224]. During late pregnancy and lactation, translocation is thought to occur more frequently due to altered tight junction regulation in the intestinal tract resulting in the efflux of immune cells to the mammary gland [225]. Furthermore, the presence of an entero-mammary circulation of IgA-producing cells is well known [226], and studies have reported that within the mesenteric lymph nodes, intestinal dendritic cells are known to retain low numbers of live commensal bacteria for a number of days [227].

Undoubtedly, anaerobes such as *Lactobacillus* and *Bifidobacterium* are transferred from mother to infant. Studies have shown that *Lactobacillus* and *Bifidobacterium* spp. isolated from mothers milk, could not be isolated from corresponding breast skin swabs, thus providing further evidence of the existence of an entero-mammary pathway [189,228]. In vitro and in vivo studies have been carried out to assess whether human milk bacteria can reach the mammary gland with the aid of dendritic cells and macrophages. Perez et al. reported internal transfer of bacteria in mice during late pregnancy and lactation. Their results suggested that bacterial translocation occurred from the gut to the mesenteric lymph nodes and mammary gland within mononuclear cells [229]. A study looking at the oral administration of three milk derived *Lactobacillus* strains (*L. salivarius* CECT 5713, *L. gasseri* CECT 5714, and *L. fermentum* CECT 5716) to treat mastitis was carried out. After probiotic treatment, two of the *Lactobacillus* strains *L. salivarius* CECT5713 and *L. fermentum* CECT5716 were detected in the milk, further elucidating the potential of an entero-mammary pathway. However, further investigations are required to determine the pathways undertaken by lactobacilli to colonise the mammary gland [230,231]. More recently, Kordy et al. used shotgun metagenomics to identify a distinct *Bifidobacterium breve* strain in the mother’s rectum, breast milk, and infant gut. Therefore, this study may support the hypothesis of entero-mammary pathway, allowing for the transport of *Bifidobacteriium breve* from maternal gut to the mammary gland [232].

## 9. Benefits of Breast Feeding

The scientific interest and benefits of the human milk microbiome is evolving, and due to advances in methodologies and research capacities, the function and role of these probiotic bacteria in maternal and infant health can be better understood. It is well reported that bacterial communities in breast milk influence overall infant health and development by seeding and shaping the gut microbiota in early life [176]. Breastfeeding molds the developing neonatal gut microbiota in early life, both directly by exposure of the newborn to the breast milk microbiota and indirectly, via maternal milk factors and bioactives that affect bacterial growth and metabolism [233].

Indeed, multiple investigations have demonstrated that breast feeding not only reduces the risk of death and disease in early life but has lasting health benefits through adult life. Breast feeding confers protection to the infant against a range of diseases such as GIT infections, necrotizing enterocolitis, respiratory tract infections, and decreases the incidence of sudden infant death syndrome. Studies have also reported breast fed infants have reduced risk of chronic diseases such as allergies, asthma, diabetes, obesity, irritable bowel syndrome, and Crohn’s disease in childhood and adult life [234,235,236,237,238,239,240,241]. Furthermore, prolonged and exclusive breast feeding has been associated with improved cognitive development in infants [242,243].

In recent years, a number of investigations have begun to explore how bacteria from human milk may function in the infant gut. The human milk microbiota has both immediate and long-term roles in reducing and preventing the incidence and severity of bacterial infections in breastfed infants by multiple mechanisms [194]. Such mechanisms include the production of antimicrobial compounds against pathogenic bacteria, competitive exclusion, prevention of adhesion of pathogenic bacteria to intestinal epithelium, and enhancing intestinal mucin production [9,10,11,244,245]. It has been documented that potentially probiotic species of *Lactobacillus* have been reported to prevent intestinal adhesion of pathogenic bacteria such as *Shigella* spp., *Salmonella* spp., and *Escherichia coli* [9,10,245,246].

The complete influence of breast milk on preterm infants is not yet fully understood; however, as the gold standard mode of nutrition, breast milk is crucial for preterm infants who are exposed to factors which disrupt development and maturation of gut bacterial communities. These factors include gestational age, birth weight, mode of delivery, antibiotic usage, and feeding regime. Preterm infants are susceptible to increased risk of necrotising enterocolitis, late onset sepsis, and mortality. Very low birth weight infants experience a very different underdeveloped gut microbiome compared to full-term infants. Gregory et al. noted that preterm infants receiving mothers own milk (MOM) appeared to mask the influence of low birth weight on the gut microbiome, and breast fed infants had a more gradual acquisition of diversity compared to formula fed infants [247]. A similar effect was seen in a study by Cong et al. where preterm infants fed MOM had an increase in gut microbial diversity over time and was constantly higher in infants fed MOM when compared to infants fed formula and donor milk [248]. It has also been reported that the gut microbiota of preterm infants exclusively breastfed resulted in increased richness and differences in microbial composition compared to preterm infants who were fed different proportions of infant formula, with formula fed infants having higher levels of *Escherichia* and *Clostridium* [249]. Although more research is needed to determine the direct health benefits of breast milk on preterm infants, feeding with breast milk appears to modulate the gut microbiota of preterm infants due to its developmental, microbial, and immune enhancing components.

## 10. Maternal Implications of Breast Feeding

As discussed previously, breastfeeding is considered the optimum feeding regime for newborn infants, playing an integral role in infant health and immunity. However, numerous factors may result in the cessation of breastfeeding and premature weaning. Inflammation of the breast tissue as a result of mastitis is one such factor. Up to 33% of lactating women are affected by mastitis [250], which is generally caused by inadequate clearing of milk from the breast resulting in infection. This results in a shift in the composition of the microbiota of the mammary gland and an increase in opportunistic pathogens [251]. Several factors determine how mastitis can be classified which include stage of lactation, clinical manifestations, and course such as acute, subacute, chronic, or recurrent [252]. Breast milk microbiota is altered in women with mastitis and a number of culture-dependent and culture-independent studies have been carried out to characterise this microbiota [251,253]. Species of the *Staphylococcus* genus are regarded as the most common cause of mastitis in breastfeeding women, with *S. aureus* identified as the main disease causing agent for acute mastitis and breast abscesses [254,255]. This is followed by *Streptococcus* spp. as the next most common cause for mastitis. Treatment for mastitis can include a course of antibiotics, however, antibiotic treatment can itself pose a number of implications, from impacting beneficial microbes in breast milk [256], thereby affecting the transfer of these microbes by means of vertical transmission to the infant as well as increasing the risk of antibiotic resistance among mastitis causing pathogens such as methicillin resistant *S. aureus* and penicillin resistance among *S. epidermidis*. Furthermore, with the threat of antibiotic resistance, alternative therapeutic approaches such as strains with probiotic potential to treat these infectious conditions must be examined.

With regards to maternal health, studies have demonstrated that ingestion of human milk derived probiotic strains *L. fermentum* CECT5716 and *L*. *salivarius* CECT5713 resulted in significant reduction in *Staphylococcus* load, reduced breast pain, faster recovery, and lower reoccurrence rates of mastitis over lactation, thus suggesting an effective alternative to antibiotics for the treatment of mastitis during lactation [231,257,258]. Further investigations documented the use of *L. fermentum* CECT5716 as a preventative measure for mastitis [259], with positives results also being reported for the use of *L. salivarius* PS2 as a preventative measure for mastitis [260].

## 11. Probiotic Bacteria in Breast Milk

Probiotics have been defined by the Food and Agriculture Organisation (FAO) and WHO as live microorganisms which when consumed in adequate amounts confer health benefits to the host [261]. Probiotic bacteria have many desirable traits, for example their ability to colonize and predominate in the neonatal gut, ability to withstand stomach acid and bile salts, adherence to the intestinal mucousa, induction of anti-inflammatory responses, inhibition of pathogens by production of antimicrobial substances, and stimulation of the immune system [9,262,263].

A number of studies have isolated potentially probiotic bacteria from human breast milk [191,222,264,265,266,267,268]. Traditionally, species of *Lactobacillus* and *Bifidobacterium* are most commonly used as probiotics in humans and have a long history of safe use. The ability of these strains to confer potential health benefits has been the subject of much analysis, and several reviews have documented their mechanisms of action [262,269]. However, investigations to isolate new probiotic strains with greater potential and higher gastrointestinal survival rate are ongoing [11]. Furthermore, it is necessary to characterise the safety and efficacy potential of probiotic strains through various in vitro tests before administering in clinical trials.

Investigations by Martin et al. led to the isolation of three *Lactobacillus* strains from milk (2 *L. gasseri* and 1 *L. fermentum*) as well as *L. salivarius* from a further study on breast milk [191,206]. *L. fermentum* and *L. salivarius* isolates underwent further investigation to determine their ability to modulate the immune system, with *L. fermentum* CECT5716 having an immunostimulatory effect in contrast to the anti-inflammatory effect of *L. salivarius* CECT5713. Ex vivo assays determined *L. fermentum* CECT5716 induced proinflammatory cytokines in rodent bone marrow derived macrophages, in contrast to the activation of IL-10 induced by *L. salivarius* CECT5713. In vivo assays in mice revealed the ingestion of *L. salivarius* CECT5713 induced IL-10 production by spleen cells, whereas ingestion of *L. fermentum* CECT5716 enhanced the production of Th1 cytokines by spleen cells and increased the IgA concentration in faeces [270].

Solís et al. isolated three *B. breve* and three *B. longum* strains from human milk, which were characterised for antimicrobial activity, GIT survival, and adherence to mucous. Although results demonstrated good probiotic potential in some of the *Bifidobacterium* strains, data from in vivo studies would be needed to conclude the true potential of isolates [265,271].

Rajoka et al. isolated seven *L. rhamnosus* strains from breast milk and assessed their ability to survive under simulated gastrointestinal conditions. Their tolerance to low pH and high bile salt concentrations were examined, with all seven isolates displaying greater than 80% survival rate at pH 2.0 and over 90% at pH 3.0 after 3 h exposure, and four isolates showed more than 80% survival rate at a bile concentration of 1.0% (*w*/*v*), indicating that they can survive passage through the digestive systems. These isolates were noted as having a higher tolerance to GIT conditions when compared to previous studies. [11] A follow up study examined three of these *L. rhamnosus* strains for their anticancer potential. The supernatant from the three strains demonstrated excellent antioxidant activity against free radicals and anticancer activity against cervix cancer cells [272].

*L. gasseri* MA-4 strain was isolated from human milk and assessed for its probiotic potential and technological properties. The strain was tested for its antimicrobial activity against a wide range of food and human pathogenic bacteria, and was shown to be effective against a number of pathogens such as *E. coli* 0157 H7, *Listeria monocytogens*, and *S*. *epidermidis. L. gasseri* MA-4 also showed high survival rates in GIT conditions and attractive technological properties due to its resistance to commonly used food additives such as nisin and sodium benzoate, thus making it a good candidate as a potential probiotic and biotherapeutic agent [273].

Although in vitro characterisation of strains to assess their health promoting effects needs to be investigated, the true probiotic potential can only be determined through animal and human studies to characterise its safety and efficacy in humans. *Lactobacillus reuteri* is a well-assessed probiotic which can be found in different parts of the body, including the skin, GIT, urinary tract, and breast milk. Some strains have been known to have benefits such as modulating the infant gut microbiota, antimicrobial activity, and ability to inhibit the colonisation of pathogenic microbes in the gut, making it an attractive therapeutic agent [274]. *L. reuteri* DSM 17938 isolated from human breast milk has been characterised and administered to infants with positive outcomes reported. Kosek et al. reported no safety concerns when administered to two to five year old infants [275]. Studies have concluded it is effective and have recommended its use for infants with colic [276,277].

Compared to vaginally delivered infants, cesarean (C)-section delivered babies have an increased abundance of *Enterobacter* but less *Bifidobacterium* in their gut microbiota. *L. reuteri* DSM 17938 was given to C-section delivered infants from two weeks to four months of age, with results showing it modulated the development of gut microbiota toward the bacterial composition in vaginally delivered infants [278]. *L. reuteri* DSM 17938 was also effective when administered to infants and children, resulting in decreased duration and frequency of acute and infectious diarrhoea [279,280,281,282] and respiratory tract infections [283].

While typically species of *Bifidobacterium* and *Lactobacillus* are most sought after for their probiotic potential, other species have been examined to determine their potential health promoting benefits. Reis et al. isolated 33 lactic acid bacteria from human breast milk, with ten belonging to *Enterooccus* spp. which displayed probiotic potential by inhibiting enteric pathogens *L. monocytogenes* and *Salmonella enterica* serotype Enteritidis, and they highlighted their ability to survive in bile salts and low pH. *Enterococcus faecium* 2C was isolated from human breast milk and was analysed for its safety and potential use in future probiotic formulations. Characterisation tests determined the strains to be non-haemolyitic and sensitive to a wide range of antibiotics. In vivo investigations in Wistar male rats showed no adverse health effects and no signs of bacteremia or infection, therefore, they may have the potential to be used in future probiotics [284]. Furthermore, Bagci et al. isolated four *E. faecium* strains from human milk and colostrum which displayed inhibitory activity against a number of pathogenic Gram-positive bacteria including *L. monocytogenes* and *S. aureus*. All isolates had the ability to survive in simulated gastrointestinal conditions and were demonstrated to be sensitive to the majority of antibiotics tested, excluding kanamycin and chloroamphenicol. The isolates did not carry antibiotic resistance genes (*vanA, vanB, er, B, tetM and aac(6′)-le-aph(2″)-la*) or possess virulence genes (*gelE, agg2, clyA, clyB, clyM*). These in vitro investigations demonstrated promising probiotic potential of the *Enterococcus* isolates, however, further in vivo studies are needed to assess and determine the safety, efficacy, and potential health benefits if used as probiotics [285].

## 12. Future of Breast Milk Research

Human breast milk is highly regarded as the optimum mode of feeding for newborns due to its ability to provide complete nutrition and capacity to confer health factors to the infant. Until recently, breast milk was considered to be a sterile fluid, however, it has now been established that breast milk has its own microbiota, which provides numerous health benefits, many of which can contribute to infant health and development.

Our understanding of the milk microbiota and its potential functions has been limited due to inability to readily cultivate many bacterial species in milk. This limitation has resulted in potential bias towards specific microorganisms which are easily culturable and these have taken the focus of most investigations. Next generation sequencing has enabled detailed insight into the complex and diverse microbial ecosystem of breast milk. These culture-independent, high-throughput sequencing methodologies have allowed better understanding of the microbiome composition. Although investigations into the breast milk microbiome using metagenomic approaches have been limited, as new methods to characterise the microbiome are developed, these approaches will enable a more comprehensive analysis of the breast milk microbiome and potential function.

However, authenticity of the human milk microbiome is subject to many factors and challenges which is common with any low biomass sample. Firstly, standardisation across milk sample collection and storage techniques are required to avoid the risk of DNA contamination from outside sources. Factors to consider with regard to sample collection include: Aseptic techniques, is breast cleaned with water before expression, and expression manually or using breast pumps devices. Although it has been shown that storage temperatures such as freezing at −80 °C had no significant impact on the microbial composition of faecal samples when compared to fresh samples, further investigations are needed to determine how storage temperature influences the microbiome of milk [286]. The addition of different commercially available preservation solutions to preserve the microbiota in milk was investigated by Lackey et al., with some solutions maintaining the bacterial communities and diversity in milk [287]. Moreover, different DNA extraction methods are shown to substantially influence the milk microbiome, showing the importance of how different methodologies introduce variability in the milk microbiome, which is apparent across many studies [204]. The composition of the breast milk microbiome is also reported to be affected by the method of 16S gene amplification used. Sequencing using different variable regions (V1-V2, V4-V5-V6, V7-V8-V9) of the 16S rDNA gene showed significant differences in the milk microbiome [205]. Furthermore, studies using mock microbial communities showed that the bacterial composition differed depending on the primers and sequencing platforms used [288]. Such results demonstrate the crucial need for standardisation across sampling and the importance of using well-defined DNA extraction protocols and sequencing technologies for the analysis of low biomass breast milk samples. In conclusion, many milk microbes are not yet characterized, and because of interstudy differences owing to the variability of sample collection, storage, processing, and analysis, risks in comparing data generated using different approaches highlight the need for standardisation.

The future role of probiotics in the infant health and nutrition market warrants further investigation. While many studies have isolated and shown health promoting effects of commensal milk derived bacteria, these have been limited to species of *Bifidobacterium* and *Lactobobacillus*. Research is needed to look outside the scope of traditional probiotic species, and the potential of next generation probiotics needs to be explored. Studies have already begun looking at gut derived next generation probiotics such as *Akkermansia municiphila* and *Faecalibacterium prausnitzii* and their potential health benefits [289,290]. This new trend looking at the potential use of non-*Bifidobacterium* and non-*Lactobacillus* commensal bacteria as probiotics to improve overall gut health has led the way to new kinds of next generation probiotics.

## References

[1] Peroni D.G., Pescollderungg L., Piacentini G.L., Rigotti E., Maselli M., Watschinger K., Piazza M., Pigozzi R., Boner A.L. (2010). Immune regulatory cytokines in the milk of lactating women from farming and urban environments. Pediatr. Allergy Immunol..

[2] Hermansson H., Kumar H., Collado M.C., Salminen S., Isolauri E., Rautava S. (2019). Breast milk microbiota is shaped by mode of delivery and intrapartum antibiotic exposure. Front. Nutr..

[3] Papachatzi E., Dimitriou G., Dimitropoulos K., Vantarakis A. (2013). Pre-pregnancy obesity: Maternal, neonatal and childhood outcomes. J. Neonatal-Perinat. Med..

[4] Kramer M.S., Guo T., Platt R.W., Sevkovskaya Z., Dzikovich I., Collet J.-P., Shapiro S., Chalmers B., Hodnett E., Vanilovich I. (2003). Infant growth and health outcomes associated with 3 compared with 6 mo of exclusive breastfeeding. Am. J. Clin. Nutr..

[5] Ladomenou F., Moschandreas J., Kafatos A., Tselentis Y., Galanakis E. (2010). Protective effect of exclusive breastfeeding against infections during infancy: A prospective study. Arch. Dis. Child..

[6] Ahern G.J., Hennessy A., Ryan C.A., Ross R.P., Stanton C. (2019). Advances in infant formula science. Annu. Rev. Food Sci. Technol..

[7] Heikkilä M.P., Saris P. (2003). Inhibition of Staphylococcus aureus by the commensal bacteria of human milk. J. Appl. Microbiol..

[8] Gavin A., Ostovar K. (1977). Microbiological characterization of human milk. J. Food Prot..

[9] Olivares M., Díaz-Ropero M., Martín R., Rodríguez J., Xaus J. (2006). Antimicrobial potential of four Lactobacillus strains isolated from breast milk. J. Appl. Microbiol..

[10] Jara S., Sánchez M., Vera R., Cofré J., Castro E. (2011). The inhibitory activity of Lactobacillus spp. isolated from breast milk on gastrointestinal pathogenic bacteria of nosocomial origin. Anaerobe.

[11] Rajoka M.S.R., Mehwish H.M., Siddiq M., Haobin Z., Zhu J., Yan L., Shao D., Xu X., Shi J. (2017). Identification, characterization, and probiotic potential of Lactobacillus rhamnosus isolated from human milk. LWT.

[12] Rodríguez J.M. (2014). The origin of human milk bacteria: Is there a bacterial entero-mammary pathway during late pregnancy and lactation?. Adv. Nutr..

[13] Javed A., Lteif A. Development of the human breast. In Seminars in Plastic Surgery.

[14] Howard B.A., Gusterson B.A. (2000). Human breast development. J. Mammary Gland Biol. Neoplasia.

[15] Seltzer V. (1994). The breast: Embryology, development, and anatomy. Clin. Obstet. Gynecol..

[16] Macias H., Hinck L. (2012). Mammary gland development. Wiley Interdiscip. Rev. Dev. Biol..

[17] Watson C.J., Khaled W.T. (2008). Mammary development in the embryo and adult: A journey of morphogenesis and commitment. Development.

[18] Gabriel A., Maxwell G. MedScape. Breast Embryology. http://emedicine.medscape.com/article/1275146-overview.

[19] Moore K.L., Persaud T.V.N., Torchia M.G. (2018). The Developing Human-E-Book: Clinically Oriented Embryology.

[20] Turashvili G., Bouchal J., Burkadze G., Kolar Z. (2005). Mammary gland development and cancer. Cesk Patol.

[21] Jayasinghe Y., Cha R., Horn-Ommen J., O’Brien P., Simmons P.S. (2010). Establishment of normative data for the amount of breast tissue present in healthy children up to two years of age. J. Pediatr. Adolesc. Gynecol..

[22] Schmidt I.M., Chellakooty M., Haavisto A.-M., Boisen K.A., Damgaard I.N., Steendahl U., Toppari J., Skakkebaek N.E., Main K.M. (2002). Gender difference in breast tissue size in infancy: Correlation with serum estradiol. Pediatr. Res..

[23] McKIERNAN J.F., Hull D. (1981). Breast development in the newborn. Arch. Dis. Child..

[24] Anbazhagan R., Bartek J., Monaghan P., Gusterson B.A. (1991). Growth and development of the human infant breast. Am. J. Anat..

[25] Naccarato A.G., Viacava P., Vignati S., Fanelli G., Bonadio A.G., Montruccoli G., Bevilacqua G. (2000). Bio-morphological events in the development of the human female mammary gland from fetal age to puberty. Virchows Arch..

[26] McNally S., Martin F. (2011). Molecular regulators of pubertal mammary gland development. Ann. Med..

[27] Marshall W.A., Tanner J.M. (1969). Variations in pattern of pubertal changes in girls. Arch. Dis. Child..

[28] Fu N.Y., Nolan E., Lindeman G.J., Visvader J.E. (2020). Stem Cells and the Differentiation Hierarchy in Mammary Gland Development. Physiol. Rev..

[29] Brisken C., Park S., Vass T., Lydon J.P., O’Malley B.W., Weinberg R.A. (1998). A paracrine role for the epithelial progesterone receptor in mammary gland development. Proc. Natl. Acad. Sci. USA.

[30] Wiseman B.S., Werb Z. (2002). Stromal effects on mammary gland development and breast cancer. Science.

[31] Richert M.M., Schwertfeger K.L., Ryder J.W., Anderson S.M. (2000). An atlas of mouse mammary gland development. J. Mammary Gland Biol. Neoplasia.

[32] Taylor-Papadimitriou J., Lane E., Neville M. (1987). The Mammary Gland: Development, Regulation and Function.

[33] Hoover K., Wilson-Clay B. (2002). The Breastfeeding Atlas.

[34] Pang W.W., Hartmann P.E. (2007). Initiation of human lactation: Secretory differentiation and secretory activation. J. Mammary Gland Biol. Neoplasia.

[35] Kent J.C. (2007). How breastfeeding works. J. Midwifery Women’s Health.

[36] Truchet S., Honvo-Houéto E. (2017). Physiology of milk secretion. Best Pract. Res. Clin. Endocrinol. Metab..

[37] Kon S.K., Cowie A.T. (2016). Milk: The Mammary Gland and Its Secretion.

[38] Pillay J., Davis T.J. (2019). Physiology, lactation. StatPearls.

[39] Neville M.C., Morton J., Umemura S. (2001). Lactogenesis: The transition from pregnancy to lactation. Pediatr. Clin. North Am..

[40] Neville M.C., Morton J. (2001). Physiology and Endocrine Changes Underlying Human Lactogenesis II. J. Nutr..

[41] Kent J.C., Mitoulas L.R., Cregan M.D., Ramsay D.T., Doherty D.A., Hartmann P.E. (2006). Volume and frequency of breastfeedings and fat content of breast milk throughout the day. Pediatrics.

[42] Hurst N.M. (2007). Recognizing and treating delayed or failed lactogenesis II. J. Midwifery Women’s Health.

[43] Howie P., Houston M., Cook A., Smart L., McArdle T., McNeilly A. (1981). How long should a breast feed last?. Early Hum. Dev..

[44] Wilde C., Prentice A., Peaker M. (1995). Breast-feeding: Matching supply with demand in human lactation. Proc. Nutr. Soc..

[45] Kent J.C., Prime D.K., Garbin C.P. (2012). Principles for maintaining or increasing breast milk production. J. Obstet. Gynecol. Neonatal Nurs..

[46] Hartmann P.E., Owens R.A., Cox D.B., Kent J.C. (1996). Breast development and control of milk synthesis. Food Nutr. Bull..

[47] Tritos N.A., Klibanski A. (2019). Prolactin and its role in human reproduction. Yen and Jaffe’s Reproductive Endocrinology.

[48] Truchet S., Ollivier-Bousquet M. (2009). Mammary gland secretion: Hormonal coordination of endocytosis and exocytosis. Animal.

[49] Mather I.H., Keenan T.W. (1998). Origin and secretion of milk lipids. J. Mammary Gland Biol. Neoplasia.

[50] Mobasheri A., Barrett-Jolley R. (2014). Aquaporin water channels in the mammary gland: From physiology to pathophysiology and neoplasia. J. Mammary Gland Biol. Neoplasia.

[51] Shennan D., Peaker M. (2000). Transport of milk constituents by the mammary gland. Physiol. Rev..

[52] Cho J.-Y., Leéveilleé R.E., Kao R., Rousset B., Parlow A., Burak W.E., Mazzaferri E.L., Jhiang S.M. (2000). Hormonal regulation of radioiodide uptake activity and Na+/I−symporter expression in mammary glands. J. Clin. Endocrinol. Metab..

[53] Montalbetti N., Dalghi M.G., Albrecht C., Hediger M.A. (2014). Nutrient transport in the mammary gland: Calcium, trace minerals and water soluble vitamins. J. Mammary Gland Biol. Neoplasia.

[54] Zhao F.-Q. (2014). Biology of glucose transport in the mammary gland. J. Mammary Gland Biol. Neoplasia.

[55] Viña J., Puertes I.R., Saez G.T., Viña J.R. (1981). Role of prolactin in amino acid uptake by the lactating mammary gland of the rat. FEBS Lett..

[56] McManaman J.L., Neville M.C. (2003). Mammary physiology and milk secretion. Adv. Drug Deliv. Rev..

[57] Kulski J., Hartmann P. (1981). Changes in human milk composition during the initiation of lactation. Aust. J. Exp. Biol. Med Sci..

[58] Kramer M.S., Kakuma R. (2012). Optimal duration of exclusive breastfeeding. Cochrane Database Syst. Rev..

[59] Casey C.E., Neifert M.R., Seacat J.M., Neville M.C. (1986). Nutrient intake by breast-fed infants during the first five days after birth. Am. J. Dis. Child..

[60] Castellote C., Casillas R., Ramírez-Santana C., Pérez-Cano F.J., Castell M., Moretones M.G., López-Sabater M.C., Franch À. (2011). Premature delivery influences the immunological composition of colostrum and transitional and mature human milk. J. Nutr..

[61] Brown J.E. (2016). Nutrition Through the Life Cycle.

[62] Pons S.M., Bargalló A.C., Folgoso C.C., Sabater M.L. (2000). Triacylglycerol composition in colostrum, transitional and mature human milk. Eur. J. Clin. Nutr..

[63] Sundekilde U.K., Downey E., O’Mahony J.A., O’Shea C.-A., Ryan C.A., Kelly A.L., Bertram H.C. (2016). The effect of gestational and lactational age on the human milk metabolome. Nutrients.

[64] Jenness R. (1979). The composition of human milk. Seminars in Perinatology.

[65] Gao X., McMahon R.J., Woo J.G., Davidson B.S., Morrow A.L., Zhang Q. (2012). Temporal changes in milk proteomes reveal developing milk functions. J. Proteome Res..

[66] D’Alessandro A., Scaloni A., Zolla L. (2010). Human milk proteins: An interactomics and updated functional overview. J. Proteome Res..

[67] Saarela T., Kokkonen J., Koivisto M. (2005). Macronutrient and energy contents of human milk fractions during the first six months of lactation. Acta Paediatr..

[68] Mitoulas L.R., Kent J.C., Cox D.B., Owens R.A., Sherriff J.L., Hartmann P.E. (2002). Variation in fat, lactose and protein in human milk over 24h and throughout the first year of lactation. Br. J. Nutr..

[69] Nasser R., Stephen A.M., Goh Y.K., Clandinin M.T. (2010). The effect of a controlled manipulation of maternal dietary fat intake on medium and long chain fatty acids in human breast milk in Saskatoon, Canada. Int. Breastfeed. J..

[70] Prentice A., Jarjou L., Drury P.J., Dewit O., Crawford M.A. (1989). Breast-milk fatty acids of rural Gambian mothers: Effects of diet and maternal parity. J. Pediatr. Gastroenterol. Nutr..

[71] Thurl S., Munzert M., Henker J., Boehm G., Müller-Werner B., Jelinek J., Stahl B. (2010). Variation of human milk oligosaccharides in relation to milk groups and lactational periods. Br. J. Nutr..

[72] Lemas D.J., Yee S., Cacho N., Miller D., Cardel M., Gurka M., Janicke D., Shenkman E. (2016). Exploring the contribution of maternal antibiotics and breastfeeding to development of the infant microbiome and pediatric obesity. Seminars in Fetal and Neonatal Medicine.

[73] Grapov D., Lemay D.G., Weber D., Phinney B.S., Azulay Chertok I.R., Gho D.S., German J.B., Smilowitz J.T. (2015). The human colostrum whey proteome is altered in gestational diabetes mellitus. J. Proteome Res..

[74] Boix-Amorós A., Collado M.C., Mira A. (2016). Relationship between milk microbiota, bacterial load, macronutrients, and human cells during lactation. Front. Microbiol..

[75] Zambruni M., Villalobos A., Somasunderam A., Westergaard S., Nigalye M., Turin C.G., Zegarra J., Bellomo S., Mercado E., Ochoa T.J. (2017). Maternal and pregnancy-related factors affecting human milk cytokines among Peruvian mothers bearing low-birth-weight neonates. J. Reprod. Immunol..

[76] Vass R.A., Kemeny A., Dergez T., Ertl T., Reglodi D., Jungling A., Tamas A. (2019). Distribution of bioactive factors in human milk samples. Int. Breastfeed. J..

[77] Ballard O., Morrow A.L. (2013). Human milk composition: Nutrients and bioactive factors. Pediatr. Clin..

[78] Smilowitz J.T., O’sullivan A., Barile D., German J.B., Lönnerdal B., Slupsky C.M. (2013). The human milk metabolome reveals diverse oligosaccharide profiles. J. Nutr..

[79] German J.B., Freeman S.L., Lebrilla C.B., Mills D.A. (2008). Human milk oligosaccharides: Evolution, structures and bioselectivity as substrates for intestinal bacteria. Personalized Nutrition for the Diverse Needs of Infants and Children.

[80] Coppa G.V., Gabrielli O., Pierani P., Catassi C., Carlucci A., Giorgi P.L. (1993). Changes in carbohydrate composition in human milk over 4 months of lactation. Pediatrics.

[81] Weichert S., Koromyslova A., Singh B.K., Hansman S., Jennewein S., Schroten H., Hansman G.S. (2016). Structural basis for norovirus inhibition by human milk oligosaccharides. J. Virol..

[82] Zhang X.-F., Tan M., Chhabra M., Dai Y.-C., Meller J., Jiang X. (2013). Inhibition of histo-blood group antigen binding as a novel strategy to block norovirus infections. PLoS ONE.

[83] Morozov V., Hansman G., Hanisch F.G., Schroten H., Kunz C. (2018). Human milk oligosaccharides as promising antivirals. Mol. Nutr. Food Res..

[84] Morrow A., Ruiz-Palacios G., Altaye M., Jiang X., Guerrero M., Meinzen-Derr J., Farkas T., Chaturvedi P., Pickering L., Newburg D. (2004). Human milk oligosaccharide blood group epitopes and innate immune protection against campylobacter and calicivirus diarrhea in breastfed infants. Protecting Infants through Human Milk.

[85] Newburg D.S., Walker W.A. (2007). Protection of the neonate by the innate immune system of developing gut and of human milk. Pediatr. Res..

[86] Ward R.E., Ninonuevo M., Mills D.A., Lebrilla C.B., German J.B. (2006). In vitro fermentation of breast milk oligosaccharides by Bifidobacterium infantis and Lactobacillus gasseri. Appl. Environ. Microbiol..

[87] Bidart G.N., Rodríguez-Díaz J., Monedero V., Yebra M.J. (2014). A unique gene cluster for the utilization of the mucosal and human milk-associated glycans galacto-N-biose and lacto-N-biose in L actobacillus casei. Mol. Microbiol..

[88] Triantis V., Bode L., Van Neerven R. (2018). Immunological effects of human milk oligosaccharides. Front. Pediatr..

[89] Marcobal A., Sonnenburg J. (2012). Human milk oligosaccharide consumption by intestinal microbiota. Clin. Microbiol. Infect..

[90] Collado M.C., Cernada M., Baüerl C., Vento M., Pérez-Martínez G. (2012). Microbial ecology and host-microbiota interactions during early life stages. Gut Microbes.

[91] Lee S.A., Lim J.Y., Kim B.-S., Cho S.J., Kim N.Y., Kim O.B., Kim Y. (2015). Comparison of the gut microbiota profile in breast-fed and formula-fed Korean infants using pyrosequencing. Nutr. Res. Pract..

[92] Vandenplas Y., Zakharova I., Dmitrieva Y. (2015). Oligosaccharides in infant formula: More evidence to validate the role of prebiotics. Br. J. Nutr..

[93] Torres D.P., Gonçalves M.D.P.F., Teixeira J.A., Rodrigues L.R. (2010). Galacto-oligosaccharides: Production, properties, applications, and significance as prebiotics. Compr. Rev. Food Sci. Food Saf..

[94] Davis L.M., Martínez I., Walter J., Goin C., Hutkins R.W. (2011). Barcoded pyrosequencing reveals that consumption of galactooligosaccharides results in a highly specific bifidogenic response in humans. PLoS ONE.

[95] Oozeer R., van Limpt K., Ludwig T., Ben Amor K., Martin R., Wind R.D., Boehm G., Knol J. (2013). Intestinal microbiology in early life: Specific prebiotics can have similar functionalities as human-milk oligosaccharides. Am. J. Clin. Nutr..

[96] Asakuma S., Hatakeyama E., Urashima T., Yoshida E., Katayama T., Yamamoto K., Kumagai H., Ashida H., Hirose J., Kitaoka M. (2011). Physiology of consumption of human milk oligosaccharides by infant gut-associated bifidobacteria. J. Biol. Chem..

[97] Marcobal A., Barboza M., Froehlich J.W., Block D.E., German J.B., Lebrilla C.B., Mills D.A. (2010). Consumption of human milk oligosaccharides by gut-related microbes. J. Agric. Food Chem..

[98] Jantscher-Krenn E., Bode L. (2012). Human milk oligosaccharides and their potential benefits for the breast-fed neonate. Minerva Pediatr..

[99] Kitaoka M. (2012). Bifidobacterial enzymes involved in the metabolism of human milk oligosaccharides. Adv. Nutr..

[100] Garrido D., Kim J.H., German J.B., Raybould H.E., Mills D.A. (2011). Oligosaccharide binding proteins from Bifidobacterium longum subsp Infantis reveal preference for host glycans. PLoS ONE.

[101] Chichlowski M., Guillaume De Lartigue J., Raybould H.E., Mills D.A. (2012). Bifidobacteria isolated from infants and cultured on human milk oligosaccharides affect intestinal epithelial function. J. Pediatr. Gastroenterol. Nutr..

[102] Wickramasinghe S., Pacheco A.R., Lemay D.G., Mills D.A. (2015). Bifidobacteria grown on human milk oligosaccharides downregulate the expression of inflammation-related genes in Caco-2 cells. BMC Microbiol..

[103] Lawson M.A., O’Neill I.J., Kujawska M., Javvadi S.G., Wijeyesekera A., Flegg Z., Chalklen L., Hall L.J. (2020). Breast milk-derived human milk oligosaccharides promote Bifidobacterium interactions within a single ecosystem. ISME J..

[104] Matsuki T., Yahagi K., Mori H., Matsumoto H., Hara T., Tajima S., Ogawa E., Kodama H., Yamamoto K., Yamada T. (2016). A key genetic factor for fucosyllactose utilization affects infant gut microbiota development. Nat. Commun..

[105] Egan M., Motherway M.O.C., Kilcoyne M., Kane M., Joshi L., Ventura M., van Sinderen D. (2014). Cross-feeding by Bifidobacterium breve UCC2003 during co-cultivation with Bifidobacterium bifidum PRL2010 in a mucin-based medium. BMC Microbiol..

[106] Egan M., Motherway M.O.C., Ventura M., van Sinderen D. (2014). Metabolism of sialic acid by Bifidobacterium breve UCC2003. Appl. Environ. Microbiol..

[107] Ward R.E., Niñonuevo M., Mills D.A., Lebrilla C.B., German J.B. (2007). In vitro fermentability of human milk oligosaccharides by several strains of bifidobacteria. Mol. Nutr. Food Res..

[108] Coulet M., Phothirath P., Allais L., Schilter B. (2014). Pre-clinical safety evaluation of the synthetic human milk, nature-identical, oligosaccharide 2′-O-Fucosyllactose (2′ FL). Regul. Toxicol. Pharmacol..

[109] Goehring K.C., Marriage B.J., Oliver J.S., Wilder J.A., Barrett E.G., Buck R.H. (2016). Similar to those who are breastfed, infants fed a formula containing 2′-fucosyllactose have lower inflammatory cytokines in a randomized controlled trial. J. Nutr..

[110] Ackerman D.L., Doster R.S., Weitkamp J.-H., Aronoff D.M., Gaddy J.A., Townsend S.D. (2017). Human milk oligosaccharides exhibit antimicrobial and antibiofilm properties against Group B Streptococcus. ACS Infect. Dis..

[111] Ackerman D.L., Craft K.M., Doster R.S., Weitkamp J.-H., Aronoff D.M., Gaddy J.A., Townsend S.D. (2017). Antimicrobial and antibiofilm activity of human milk oligosaccharides against Streptococcus agalactiae, Staphylococcus aureus, and Acinetobacter baumannii. ACS Infect. Dis..

[112] Chambers S.A., Townsend S.D. (2020). Bioorthogonal human milk oligosaccharide probes for antimicrobial target identification within Streptococcus agalactiae. Carbohydr. Res..

[113] Bushati N., Cohen S.M. (2007). MicroRNA functions. Annu. Rev. Cell Dev. Biol..

[114] Alvarez-Garcia I., Miska E.A. (2005). MicroRNA functions in animal development and human disease. Development.

[115] Wienholds E., Plasterk R.H. (2005). MicroRNA function in animal development. FEBS Lett..

[116] Kittelmann S., McGregor A.P. (2019). Modulation and evolution of animal development through microRNA regulation of gene expression. Genes.

[117] Tsukamoto M., Iinuma H., Matsuda K., Yagi T., Hashiguchi Y. (2017). Circulating exosomal microRNA-21 as a biomarker in each tumor stage of colorectal cancer. Oncology.

[118] Vu T.L., Peng B., Zhang D.X., Ma V., Mathey-Andrews C.A., Lam C.K., Kiomourtzis T., Jin J., McReynolds L., Huang L. (2019). Tumor-secreted extracellular vesicles promote the activation of cancer-associated fibroblasts via the transfer of microRNA-125b. J. Extracell. Vesicles.

[119] Kim S., Lee E., Jung J., Lee J.W., Kim H.J., Kim J., Yoo H.J., Lee H.J., Chae S.Y., Jeon S.-M. (2018). microRNA-155 positively regulates glucose metabolism via PIK3R1-FOXO3a-cMYC axis in breast cancer. Oncogene.

[120] Weber J.A., Baxter D.H., Zhang S., Huang D.Y., How Huang K., Jen Lee M., Galas D.J., Wang K. (2010). The microRNA spectrum in 12 body fluids. Clin. Chem..

[121] Liao Y., Du X., Li J., Lönnerdal B. (2017). Human milk exosomes and their microRNAs survive digestion in vitro and are taken up by human intestinal cells. Mol. Nutr. Food Res..

[122] Wang J., Chen J., Sen S. (2016). MicroRNA as biomarkers and diagnostics. J. Cell. Physiol..

[123] Gallo A., Tandon M., Alevizos I., Illei G.G. (2012). The majority of microRNAs detectable in serum and saliva is concentrated in exosomes. PLoS ONE.

[124] Zhou Q., Li M., Wang X., Li Q., Wang T., Zhu Q., Zhou X., Wang X., Gao X., Li X. (2012). Immune-related microRNAs are abundant in breast milk exosomes. Int. J. Biol. Sci..

[125] Alsaweed M., Lai C.T., Hartmann P.E., Geddes D.T., Kakulas F. (2016). Human milk cells and lipids conserve numerous known and novel miRNAs, some of which are differentially expressed during lactation. PLoS ONE.

[126] Alsaweed M., Lai C.T., Hartmann P.E., Geddes D.T., Kakulas F. (2016). Human milk miRNAs primarily originate from the mammary gland resulting in unique miRNA profiles of fractionated milk. Sci. Rep..

[127] Alsaweed M., Hepworth A.R., Lefevre C., Hartmann P.E., Geddes D.T., Hassiotou F. (2015). Human milk microRNA and total RNA differ depending on milk fractionation. J. Cell. Biochem..

[128] Carney M.C., Tarasiuk A., DiAngelo S.L., Silveyra P., Podany A., Birch L.L., Paul I.M., Kelleher S., Hicks S.D. (2017). Metabolism-related microRNAs in maternal breast milk are influenced by premature delivery. Pediatr. Res..

[129] Shiff Y.E., Reif S., Marom R., Shiff K., Reifen R., Golan-Gerstl R. (2019). MiRNA-320a is less expressed and miRNA-148a more expressed in preterm human milk compared to term human milk. J. Funct. Foods.

[130] Alsaweed M., Hartmann P.E., Geddes D.T., Kakulas F. (2015). MicroRNAs in breastmilk and the lactating breast: Potential immunoprotectors and developmental regulators for the infant and the mother. Int. J. Environ. Res. Public Health.

[131] Kosaka N., Izumi H., Sekine K., Ochiya T. (2010). microRNA as a new immune-regulatory agent in breast milk. Silence.

[132] Chu M., Zhao Y., Yu S., Hao Y., Zhang P., Feng Y., Zhang H., Ma D., Liu J., Cheng M. (2018). MicroRNA-221 may be involved in lipid metabolism in mammary epithelial cells. Int. J. Biochem. Cell Biol..

[133] Aly E., Darwish A.A., Lopez-Nicolas R., Frontela-Saseta C., Ros-Berruezo G. (2018). Bioactive Components of Human Milk: Similarities and Differences between Human Milk and Infant Formula. Selected Topics in Breastfeeding.

[134] Vogel H.J. (2012). Lactoferrin, a bird’s eye view. Biochem. Cell Biol..

[135] Ochoa T.J., Chea-Woo E., Baiocchi N., Pecho I., Campos M., Prada A., Valdiviezo G., Lluque A., Lai D., Cleary T.G. (2013). Randomized double-blind controlled trial of bovine lactoferrin for prevention of diarrhea in children. J. Pediatr..

[136] Manzoni P., Rinaldi M., Cattani S., Pugni L., Romeo M.G., Messner H., Stolfi I., Decembrino L., Laforgia N., Vagnarelli F. (2009). Bovine lactoferrin supplementation for prevention of late-onset sepsis in very low-birth-weight neonates: A randomized trial. JAMA.

[137] Manzoni P., Sánchez R.G., Meyer M., Stolfi I., Pugni L., Messner H., Cattani S., Betta P.M., Memo L., Decembrino L. (2018). Exposure to gastric acid inhibitors increases the risk of infection in preterm very low birth weight infants but concomitant administration of lactoferrin counteracts this effect. J. Pediatr..

[138] Chang C.-J., Chao J.C.-J. (2002). Effect of human milk and epidermal growth factor on growth of human intestinal Caco-2 cells. J. Pediatr. Gastroenterol. Nutr..

[139] Hirai C., Ichiba H., Saito M., Shintaku H., Yamano T., Kusuda S. (2002). Trophic effect of multiple growth factors in amniotic fluid or human milk on cultured human fetal small intestinal cells. J. Pediatr. Gastroenterol. Nutr..

[140] Boesmans W., Gomes P., Janssens J., Tack J., Berghe P.V. (2008). Brain-derived neurotrophic factor amplifies neurotransmitter responses and promotes synaptic communication in the enteric nervous system. Gut.

[141] Blum J., Baumrucker C. (2002). Colostral and milk insulin-like growth factors and related substances: Mammary gland and neonatal (intestinal and systemic) targets. Domest. Anim. Endocrinol..

[142] Hurley W.L., Theil P.K. (2011). Perspectives on immunoglobulins in colostrum and milk. Nutrients.

[143] Lawrence R.M., Lawrence R.A. (2004). Breast milk and infection. Clin. Perinatol..

[144] Hanson L.Å., Korotkova M. (2002). The role of breastfeeding in prevention of neonatal infection. Seminars in Neonatology.

[145] Lucas A. (2005). Long-term programming effects of early nutrition-Implications for the preterm infant. J. Perinatol..

[146] Hylander M.A., Strobino D.M., Dhanireddy R. (1998). Human milk feedings and infection among very low birth weight infants. Pediatrics.

[147] Parkinson J.R., Hyde M.J., Gale C., Santhakumaran S., Modi N. (2013). Preterm birth and the metabolic syndrome in adult life: A systematic review and meta-analysis. Pediatrics.

[148] Sullivan S., Schanler R.J., Kim J.H., Patel A.L., Trawöger R., Kiechl-Kohlendorfer U., Chan G.M., Blanco C.L., Abrams S., Cotten C.M. (2010). An exclusively human milk-based diet is associated with a lower rate of necrotizing enterocolitis than a diet of human milk and bovine milk-based products. J. Pediatr..

[149] Patel P., Bhatia J. (2016). Human Milk: The Preferred First Food for Premature Infants. J Hum Nutr Food Sci.

[150] Tudehope D.I. (2013). Human milk and the nutritional needs of preterm infants. J. Pediatr..

[151] Bauer J., Gerss J. (2011). Longitudinal analysis of macronutrients and minerals in human milk produced by mothers of preterm infants. Clin. Nutr..

[152] De Figueiredo C.S.M., Palhares D.B., Melnikov P., Moura A.J.d.C.M., dos Santos S.C. (2010). Zinc and copper concentrations in human preterm milk. Biol. Trace Elem. Res..

[153] O’Brien C.E., Krebs N.F., Westcott J.L., Dong F. (2007). Relationships among plasma zinc, plasma prolactin, milk transfer, and milk zinc in lactating women. J. Hum. Lact..

[154] Dvorak B., Fituch C.C., Williams C.S., Hurst N.M., Schanler R.J. (2003). Increased epidermal growth factor levels in human milk of mothers with extremely premature infants. Pediatr. Res..

[155] Gross S.J., Buckley R.H., Wakil S.S., McAllister D.C., David R.J., Faix R.G. (1981). Elevated IgA concentration in milk produced by mothers delivered of preterm infants. J. Pediatr..

[156] Kunz C., Kuntz S., Rudloff S., Moreno F., Sanz M. (2014). Food Oligosaccharides: Production, Analysis and Bioactivity.

[157] Montagne P., Cuillière M.L., Molé C., Béné M.C., Faure G. (1999). Immunological and nutritional composition of human milk in relation to prematurity and mothers’ parity during the first 2 weeks of lactation. J. Pediatr. Gastroenterol. Nutr..

[158] Arora S., McJunkin C., Wehrer J., Kuhn P. (2000). Major factors influencing breastfeeding rates: Mother’s perception of father’s attitude and milk supply. Pediatrics.

[159] Kozhimannil K.B., Jou J., Attanasio L.B., Joarnt L.K., McGovern P. (2014). Medically complex pregnancies and early breastfeeding behaviors: A retrospective analysis. PLoS ONE.

[160] Carver J.D. (2003). Advances in nutritional modifications of infant formulas. Am. J. Clin. Nutr..

[161] Grant C., Rotherham B., Sharpe S., Scragg R., Thompson J., Andrews J., Wall C., Murphy J., Lowry D. (2005). Randomized, double-blind comparison of growth in infants receiving goat milk formula versus cow milk infant formula. J. Paediatr. Child Health.

[162] Martin C.R., Ling P.-R., Blackburn G.L. (2016). Review of infant feeding: Key features of breast milk and infant formula. Nutrients.

[163] Cook D.A. (1989). Nutrient levels in infant formulas: Technical considerations. J. Nutr..

[164] Thompkinson D., Kharb S. (2007). Aspects of infant food formulation. Compr. Rev. Food Sci. Food Saf..

[165] Koletzko B., Baker S., Cleghorn G., Neto U.F., Gopalan S., Hernell O., Hock Q.S., Jirapinyo P., Lonnerdal B., Pencharz P. (2005). Global standard for the composition of infant formula: Recommendations of an ESPGHAN coordinated international expert group. J. Pediatr. Gastroenterol. Nutr..

[166] Koletzko B., Broekaert I., Demmelmair H., Franke J., Hannibal I., Oberle D., Schiess S., Baumann B.T., Verwied-Jorky S. (2005). Protein intake in the first year of life: A risk factor for later obesity?. Early Nutrition and Its Later Consequences: New Opportunities.

[167] Høst A., Halken S., Jacobsen H.P., Christensen A.E., Herskind A.M., Plesner K. (2002). Clinical course of cow’s milk protein allergy/intolerance and atopic diseases in childhood. Pediat. Allergy Immunol..

[168] Arslanoglu S., Moro G.E., Boehm G. (2007). Early supplementation of prebiotic oligosaccharides protects formula-fed infants against infections during the first 6 months of life. J. Nutr..

[169] Moro G., Arslanoglu S., Stahl B., Jelinek J., Wahn U., Boehm G. (2006). A mixture of prebiotic oligosaccharides reduces the incidence of atopic dermatitis during the first six months of age. Arch. Dis. Child..

[170] Grüber C., Van Stuijvenberg M., Mosca F., Moro G., Chirico G., Braegger C.P., Riedler J., Boehm G., Wahn U., Group M.W. (2010). Reduced occurrence of early atopic dermatitis because of immunoactive prebiotics among low-atopy-risk infants. J. Allergy Clin. Immunol..

[171] Martín R., Langa S., Reviriego C., Jimínez E., Marín M.L., Xaus J., Fernández L., Rodríguez J.M. (2003). Human milk is a source of lactic acid bacteria for the infant gut. J. Pediatr..

[172] Ward T.L., Hosid S., Ioshikhes I., Altosaar I. (2013). Human milk metagenome: A functional capacity analysis. BMC Microbiol..

[173] Aakko J., Kumar H., Rautava S., Wise A., Autran C., Bode L., Salminen S. (2017). Human milk oligosaccharide categories define the microbiota composition in human colostrum. Benef. Microbes.

[174] Murphy K., Curley D., O’Callaghan T.F., O’Shea C.-A., Dempsey E.M., O’Toole P.W., Ross R.P., Ryan C.A., Stanton C. (2017). The composition of human milk and infant faecal microbiota over the first three months of life: A pilot study. Sci. Rep..

[175] Lackey K.A., Williams J.E., Meehan C.L., Zachek J.A., Benda E.D., Price W.J., Foster J.A., Sellen D.W., Kamau-Mbuthia E.W., Kamundia E.W. (2019). What’s normal? microbiomes in human milk and infant feces are related to each other but vary geographically: The INSPIRE study. Front. Nutr..

[176] Pannaraj P.S., Li F., Cerini C., Bender J.M., Yang S., Rollie A., Adisetiyo H., Zabih S., Lincez P.J., Bittinger K. (2017). Association Between Breast Milk Bacterial Communities and Establishment and Development of the Infant Gut Microbiome. JAMA Pediatr..

[177] Eidelman A.I., Szilagyi G. (1979). Patterns of bacterial colonization of human milk. Obstet. Gynecol..

[178] Osterman K.L., Rahm V.-A. (2000). Lactation Mastitis: Bacterial Cultivation of Breast Milk, Symptoms, Treatment, and Outcome. J. Hum. Lact..

[179] Thomsen A.C., Espersen T., Maigaard S. (1984). Course and treatment of milk stasis, noninfectious inflammation of the breast, and infectious mastitis in nursing women. Am. J. Obstet. Gynecol..

[180] Jones C. (2001). Maternal transmission of infectious pathogens in breast milk. J. Paediatr. Child Health.

[181] Kumar A., Yadav M., Kakkar S. (1981). Human milk as a source of Q-fever infection in breast-fed babies. Indian J. Med. Res..

[182] Le Thomas I., Mariani-Kurkdjian P., Collignon A., Gravet A., Clermont O., Brahimi N.m., Gaudelus J., Aujard Y., Navarro J., Beaufils F. (2001). Breast milk transmission of a Panton-Valentine leukocidin-producing Staphylococcus aureus strain causing infantile pneumonia. J. Clin. Microbiol..

[183] Ryder R.W., Crosby-Ritchie A., McDonough B., Hall W.J. (1977). Human milk contaminated with Salmonella kottbus: A cause of nosocomial illness in infants. JAMA.

[184] Botsford K.B., Weinstein R.A., Boyer K.M., Nathan C., Carman M., Paton J.B. (1986). Gram-negative bacilli in human milk feedings: Quantitation and clinical consequences for premature infants. J. Pediatr..

[185] Larson E., Zuill R., Zier V., Berg B. (1984). Storage of human breast milk. Infect. Control Hosp. Epidemiol..

[186] Sosa R., Barness L. (1987). Bacterial Growth in Refrigerated Human Milk. Am. J. Dis. Child..

[187] Jost T., Lacroix C., Braegger C., Chassard C. (2013). Assessment of bacterial diversity in breast milk using culture-dependent and culture-independent approaches. Br. J. Nutr..

[188] Sinkiewicz G., Nordström E.A. (2005). 353 occurrence of Lactobacillus reuteri, lactobacilli and bifidobacteria in human breast milk. Pediatric Res..

[189] Martín R., Jiménez E., Heilig H., Fernández L., Marín M.L., Zoetendal E.G., Rodríguez J.M. (2009). Isolation of Bifidobacteria from Breast Milk and Assessment of the Bifidobacterial Population by PCR-Denaturing Gradient Gel Electrophoresis and Quantitative Real-Time PCR. Appl. Environ. Microbiol..

[190] Kansandee W., Moonmangmee D., Moonmangmee S., Itsaranuwat P. (2019). Characterization and Bifidobacterium sp. growth stimulation of exopolysaccharide produced by Enterococcus faecalis EJRM152 isolated from human breast milk. Carbohydr. Polym..

[191] Martín R., Olivares M., Marín M.L., Fernández L., Xaus J., Rodríguez J.M. (2005). Probiotic potential of 3 lactobacilli strains isolated from breast milk. J. Hum. Lact..

[192] Belhadj F.Z.B., Boublenza F., Karam N.-E. (2020). Stress tolerance in Lactobacillus plantarum LMF6 isolated from human breast milk. South Asian J. Exp. Biol..

[193] Li N., Pang B., Liu G., Zhao X., Xu X., Jiang C., Yang B., Liu Y., Shi J. (2020). Lactobacillus rhamnosus from human breast milk shows therapeutic function against foodborne infection by multi-drug resistant Escherichia coli in mice. Food Funct..

[194] Le Doare K., Holder B., Bassett A., Pannaraj P.S. (2018). Mother’s milk: A purposeful contribution to the development of the infant microbiota and immunity. Front. Immunol..

[195] Hunt K.M., Foster J.A., Forney L.J., Schütte U.M., Beck D.L., Abdo Z., Fox L.K., Williams J.E., McGuire M.K., McGuire M.A. (2011). Characterization of the diversity and temporal stability of bacterial communities in human milk. PLoS ONE.

[196] Chen P.-W., Lin Y.-L., Huang M.-S. (2018). Profiles of commensal and opportunistic bacteria in human milk from healthy donors in Taiwan. J. Food Drug Anal..

[197] Li S.-W., Watanabe K., Hsu C.-C., Chao S.-H., Yang Z.-H., Lin Y.-J., Chen C.-C., Cao Y.-M., Huang H.-C., Chang C.-H. (2017). Bacterial composition and diversity in breast milk samples from mothers living in Taiwan and mainland China. Front. Microbiol..

[198] Fernández L., Browne P.D., Aparicio M., Alba C., Hechler C., Beijers R., Rodríguez J.M., de Weerth C. (2019). Human milk microbiome and maternal postnatal psychosocial distress. Front. Microbiol..

[199] Cabrera-Rubio R., Collado M.C., Laitinen K., Salminen S., Isolauri E., Mira A. (2012). The human milk microbiome changes over lactation and is shaped by maternal weight and mode of delivery. Am. J. Clin. Nutr..

[200] Khodayar-Pardo P., Mira-Pascual L., Collado M.C., Martínez-Costa C. (2014). Impact of lactation stage, gestational age and mode of delivery on breast milk microbiota. J. Perinatol..

[201] Soto A., Martín V., Jiménez E., Mader I., Rodríguez J.M., Fernández L. (2014). Lactobacilli and bifidobacteria in human breast milk: Influence of antibiotherapy and other host and clinical factors. J. Pediatr. Gastroenterol. Nutr..

[202] Olivares M., Albrecht S., De Palma G., Ferrer M.D., Castillejo G., Schols H.A., Sanz Y. (2015). Human milk composition differs in healthy mothers and mothers with celiac disease. Eur. J. Nutr..

[203] Kumar H., du Toit E., Kulkarni A., Aakko J., Linderborg K.M., Zhang Y., Nicol M.P., Isolauri E., Yang B., Collado M.C. (2016). Distinct Patterns in Human Milk Microbiota and Fatty Acid Profiles Across Specific Geographic Locations. Front. Microbiol..

[204] Douglas C.A., Ivey K.L., Papanicolas L.E., Best K.P., Muhlhausler B.S., Rogers G.B. (2020). DNA extraction approaches substantially influence the assessment of the human breast milk microbiome. Sci. Rep..

[205] Meyer K.M., Pace R.M., Mohammad M., Haymond M., Aagaard K.M. (2019). 941: Composition of the breast milk microbiome is influenced by the method of 16S-amplicon sequencing used. Am. J. Obstet. Gynecol..

[206] Martín R., Jiménez E., Olivares M., Marín M., Fernández L., Xaus J., Rodríguez J. (2006). Lactobacillus salivarius CECT 5713, a potential probiotic strain isolated from infant feces and breast milk of a mother–child pair. Int. J. Food Microbiol..

[207] Albesharat R., Ehrmann M.A., Korakli M., Yazaji S., Vogel R.F. (2011). Phenotypic and genotypic analyses of lactic acid bacteria in local fermented food, breast milk and faeces of mothers and their babies. Syst. Appl. Microbiol..

[208] Makino H., Kushiro A., Ishikawa E., Muylaert D., Kubota H., Sakai T., Oishi K., Martin R., Amor K.B., Oozeer R. (2011). Transmission of intestinal Bifidobacterium longum subsp. longum strains from mother to infant, determined by multilocus sequencing typing and amplified fragment length polymorphism. Appl. Environ. Microbiol..

[209] Martín V., Maldonado-Barragán A., Moles L., Rodriguez-Baños M., Campo R.d., Fernández L., Rodríguez J.M., Jiménez E. (2012). Sharing of bacterial strains between breast milk and infant feces. J. Hum. Lact..

[210] Eshaghi M., Bibalan M.H., Rohani M., Esghaei M., Douraghi M., Talebi M., Pourshafie M.R. (2017). Bifidobacterium obtained from mother’s milk and their infant stool; A comparative genotyping and antibacterial analysis. Microb. Pathog..

[211] Urbaniak C., Angelini M., Gloor G.B., Reid G. (2016). Human milk microbiota profiles in relation to birthing method, gestation and infant gender. Microbiome.

[212] Biagi E., Aceti A., Quercia S., Beghetti I., Rampelli S., Turroni S., Soverini M., Zambrini A.V., Faldella G., Candela M. (2018). Microbial Community Dynamics in Mother’s Milk and Infant’s Mouth and Gut in Moderately Preterm Infants. Front. Microbiol..

[213] Dimitriu P.A., Iker B., Malik K., Leung H., Mohn W., Hillebrand G.G. (2019). New insights into the intrinsic and extrinsic factors that shape the human skin microbiome. MBio.

[214] Ramsay D.T., Kent J.C., Owens R.A., Hartmann P.E. (2004). Ultrasound imaging of milk ejection in the breast of lactating women. Pediatrics.

[215] Williams J.E., Carrothers J.M., Lackey K.A., Beatty N.F., Brooker S.L., Peterson H.K., Steinkamp K.M., York M.A., Shafii B., Price W.J. (2019). Strong multivariate relations exist among milk, oral, and fecal microbiomes in mother-infant dyads during the first six months postpartum. J. Nutr..

[216] Geddes D.T. (2009). The use of ultrasound to identify milk ejection in women–tips and pitfalls. Int. Breastfeed. J..

[217] Moossavi S., Sepehri S., Robertson B., Bode L., Goruk S., Field C.J., Lix L.M., de Souza R.J., Becker A.B., Mandhane P.J. (2019). Composition and variation of the human milk microbiota are influenced by maternal and early-life factors. Cell Host Microbe.

[218] Moossavi S., Azad M.B. (2019). Origins of human milk microbiota: New evidence and arising questions. Gut Microbes.

[219] Riskin A., Almog M., Peri R., Halasz K., Srugo I., Kessel A. (2012). Changes in immunomodulatory constituents of human milk in response to active infection in the nursing infant. Pediatr. Res..

[220] Hassiotou F., Hepworth A.R., Metzger P., Tat Lai C., Trengove N., Hartmann P.E., Filgueira L. (2013). Maternal and infant infections stimulate a rapid leukocyte response in breastmilk. Clin. Transl. Immunol..

[221] Martén R.O., Langa S., Reviriego C., Jiménez E., Martǩn M.A.L., Olivares M., Boza J., Jiménez J., Fernández L., Xaus J. (2004). The commensal microflora of human milk: New perspectives for food bacteriotherapy and probiotics. Trends Food Sci. Technol..

[222] Damaceno Q.S., Souza J.P., Nicoli J.R., Paula R.L., Assis G.B., Figueiredo H.C., Azevedo V., Martins F.S. (2017). Evaluation of potential probiotics isolated from human milk and colostrum. Probiotics Antimicrob. Proteins.

[223] Rescigno M., Urbano M., Valzasina B., Francolini M., Rotta G., Bonasio R., Granucci F., Kraehenbuhl J.-P., Ricciardi-Castagnoli P. (2001). Dendritic cells express tight junction proteins and penetrate gut epithelial monolayers to sample bacteria. Nat. Immunol..

[224] Langa S., Maldonado-Barragán A., Delgado S., Martín R., Martín V., Jiménez E., Ruíz-Barba J.L., Mayo B., Connor R.I., Suárez J.E. (2012). Characterization of Lactobacillus salivarius CECT 5713, a strain isolated from human milk: From genotype to phenotype. Appl. Microbiol. Biotechnol..

[225] Fernández L., Langa S., Martín V., Maldonado A., Jiménez E., Martín R., Rodríguez J.M. (2013). The human milk microbiota: Origin and potential roles in health and disease. Pharmacol. Res..

[226] Newburg D.S. (2005). Innate immunity and human milk. J. Nutr..

[227] Macpherson A.J., Uhr T. (2004). Induction of protective IgA by intestinal dendritic cells carrying commensal bacteria. Science.

[228] Gueimonde M., Laitinen K., Salminen S., Isolauri E. (2007). Breast milk: A source of bifidobacteria for infant gut development and maturation?. Neonatology.

[229] Perez P.F., Doré J., Leclerc M., Levenez F., Benyacoub J., Serrant P., Segura-Roggero I., Schiffrin E.J., Donnet-Hughes A. (2007). Bacterial imprinting of the neonatal immune system: Lessons from maternal cells?. Pediatrics.

[230] Jiménez E., Fernández L., Maldonado A., Martín R., Olivares M., Xaus J., Rodríguez J. (2008). Oral administration of Lactobacillus strains isolated from breast milk as an alternative for the treatment of infectious mastitis during lactation. Appl. Env. Microbiol..

[231] Arroyo R., Martín V., Maldonado A., Jiménez E., Fernández L., Rodríguez J.M. (2010). Treatment of Infectious Mastitis during Lactation: Antibiotics versus Oral Administration of Lactobacilli Isolated from Breast Milk. Clin. Infect. Dis..

[232] Kordy K., Gaufin T., Mwangi M., Li F., Cerini C., Lee D.J., Adisetiyo H., Woodward C., Pannaraj P.S., Tobin N.H. (2020). Contributions to human breast milk microbiome and enteromammary transfer of Bifidobacterium breve. PLoS ONE.

[233] Elsen L.V.D., Garssen J., Burcelin R., Verhasselt V. (2019). Shaping the gut microbiota by breastfeeding: the gateway to allergy prevention?. Front. Pediatr..

[234] Eidelman A.I., Schanler R.J. (2012). Breastfeeding and the use of human milk. Pediatrics.

[235] Parikh N., Hwang S.J., Ingelsson E., Benjamin E.J., Fox C.S., Vasan R.S., Murabito J.M. (2009). Breastfeeding in infancy and adult cardiovascular disease risk factors. Am. J. Med..

[236] Wang L., Collins C., Ratliff M., Xie B., Wang Y. (2017). Breastfeeding reduces childhood obesity risks. Child. Obes..

[237] Klopp A., Vehling L., Becker A.B., Subbarao P., Mandhane P.J., E Turvey S., Lefebvre D.L., Sears M.R., Azad M.B., Daley D. (2017). Modes of infant feeding and the risk of childhood asthma: A prospective birth cohort study. J. Pediatr..

[238] Xu L., Lochhead P., Ko Y., Claggett B., Leong R.W., Ananthakrishnan A.N. (2017). Systematic review with meta-analysis: Breastfeeding and the risk of Crohn’s disease and ulcerative colitis. Aliment. Pharmacol. Ther..

[239] Dogaru C., Nyffenegger D., Pescatore A.M., Spycher B.D., E Kuehni C. (2014). Breastfeeding and childhood asthma: Systematic review and meta-analysis. Am. J. Epidemiology.

[240] Horta B.L., De Mola C.L., Victora C.G. (2015). Long-term consequences of breastfeeding on cholesterol, obesity, systolic blood pressure and type 2 diabetes: A systematic review and meta-analysis. Acta Paediatr..

[241] Temples H.S. (2019). Breastfeeding reduces risk of Type 2 Diabetes in the (PETS). Nurs. Outlook.

[242] Michaelsen F.K., Lauritzen L., Mortensen E.L. (2009). Effects of Breast-feeding on Cognitive Function. Breast-Feeding: Early Influences on Later Health.

[243] Ibrahim H.S., El-Ghany A., Mohamed S., El Shafie M.T., Hady E. (2019). Cognitive Functions in Breastfed versus Artificially Fed in Preschool Children. Egypt. J. Hosp. Med..

[244] Mackie R.I., Sghir A., Gaskins H.R. (1999). Developmental microbial ecology of the neonatal gastrointestinal tract. Am. J. Clin. Nutr..

[245] Lyons A., O’Mahony D., O’Brien F., Mac Sharry J., Sheil B., Ceddia M., Russell W.M., Forsythe P., Bienenstock J., Kiely B. (2010). Bacterial strain-specific induction of Foxp3+ T regulatory cells is protective in murine allergy models. Clin. Exp. Allergy.

[246] Nantavisai K., Puttikamonkul S., Chotelersak K., Taweechotipatr M. (2018). In vitro adhesion property and competition against enteropathogens of Lactobacillus strains isolated from Thai infants. Songklanakarin J. Sci. Technol..

[247] Gregory K.E., Samuel B., Houghteling P., Shan G., Ausubel F., Sadreyev R.I., Walker W.A. (2016). Influence of maternal breast milk ingestion on acquisition of the intestinal microbiome in preterm infants. Microbiome.

[248] Cong X., Judge M., Xu W., Diallo A., Janton S., Brownell E.A., Maas K., Graf J. (2017). Influence of infant feeding type on gut microbiome development in hospitalized preterm infants. Nurs. Res..

[249] Zanella A., Silveira R.C., Roesch L.F.W., Corso A.L., Dobbler P.T., Mai V., Procianoy R.S. (2019). Influence of own mother’s milk and different proportions of formula on intestinal microbiota of very preterm newborns. PLoS ONE.

[250] Inch S., Von Xylander S. (2000). Mastitis: Causes and Management.

[251] Patel S., Vaidya Y.H., Patel R., Pandit R.J., Joshi C., Kunjadiya A. (2017). Culture independent assessment of human milk microbial community in lactational mastitis. Sci. Rep..

[252] Contreras G.A., Rodríguez J.M. (2011). Mastitis: Comparative Etiology and Epidemiology. J. Mammary Gland. Boil. Neoplasia.

[253] Patel S., Vaidya Y.H., Joshi C., Kunjadia A.P. (2015). Culture-dependent assessment of bacterial diversity from human milk with lactational mastitis. Comp. Haematol. Int..

[254] Moazzez A. (2007). Breast abscess bacteriologic features in the era of community-acquired methicillin-resistant Staphylococcus aureus epidemics. Arch. Surg..

[255] Branch-Elliman W., Lee G.M., Golen T.H., Gold H.S., Baldini L.M., Wright S.B. (2013). Health and economic burden of post-partum Staphylococcus aureus breast abscess. PLoS ONE.

[256] Padilha M., Iaucci J., Cabral V., Diniz E., Taddei C., Saad S.M.I. (2019). Maternal antibiotic prophylaxis affects Bifidobacterium spp. counts in the human milk, during the first week after delivery. Benef. Microbes.

[257] Maldonado-Lobón J.A., Díaz-López M.A., Carputo R., Duarte P., Díaz-Ropero M.P., Valero A.D., Sañudo A., Sempere L., Ruiz-López M.D., Bañuelos O. (2015). Lactobacillus fermentum CECT 5716 reduces Staphylococcus load in the breastmilk of lactating mothers suffering breast pain: A randomized controlled trial. Breastfeed. Med..

[258] Hurtado J.A., Maldonado-Lobón J.A., Díaz-Ropero M.P., Flores-Rojas K., Uberos J., Leante J.L., Affumicato L., Couce M.L., Garrido J.M., Olivares M. (2017). Oral administration to nursing women of Lactobacillus fermentum CECT5716 prevents lactational mastitis development: A randomized controlled trial. Breastfeed. Med..

[259] Bond D.M., Morris J.M., Nassar N. (2017). Nassar, Study protocol: evaluation of the probiotic Lactobacillus Fermentum CECT5716 for the prevention of mastitis in breastfeeding women: A randomised controlled trial. BMC Pregnancy Childbirth.

[260] Fernández L., Cárdenas N., Arroyo R., Manzano S., Jiménez E., Martin V., Rodríguez J.M. (2015). Prevention of infectious mastitis by oral administration of Lactobacillus salivarius PS2 during late pregnancy. Clin. Infect. Dis..

[261] Hill C., Guarner F., Reid G., Gibson G.R., Merenstein D.J., Pot B., Calder P.C. (2014). Expert consensus document: The International Scientific Association for Probiotics and Prebiotics consensus statement on the scope and appropriate use of the term probiotic. Nat. Rev. Gastroenterol. Hepatol..

[262] Diaz J.P., Ruiz-Ojeda F.J., Gil-Campos M. (2019). Mechanisms of action of probiotic. Adv. Nutr..

[263] Yousefi B., Eslami M., Ghasemian A., Kokhaei P., Farrokhi A.S., Darabi N. (2018). Probiotics importance and their immunomodulatory properties. J. Cell. Physiol..

[264] Lara-Villoslada F., Olivares M., Sierra S., Rodríguez J.M., Boza J., Xaus J. (2007). Beneficial effects of probiotic bacteria isolated from breast milk. Br. J. Nutr..

[265] Arboleya S., Ruas-Madiedo P., Margolles A., Solís G., Salminen S., Reyes-Gavilán C.G.D.L., Gueimonde M. (2011). Characterization and in vitro properties of potentially probiotic Bifidobacterium strains isolated from breast-milk. Int. J. Food Microbiol..

[266] Mehanna N.S., Tawfik N.F., Salem M.M., Effat B.A., Gad El-Rab D.A. (2013). Assessment of potential probiotic bacteria isolated from breast milk. Middle East J. Sci. Res..

[267] Kozak K., Charbonneau D.L., Sanozky-Dawes R., Klaenhammer T. (2016). Characterization of bacterial isolates from the microbiota of mothers’ breast milk and their infants. Gut Microbes.

[268] Jamyuang C., Phoonlapdacha P., Chongviriyaphan N., Chanput W., Nitisinprasert S., Nakphaichit M. (2019). Characterization and probiotic properties of Lactobacilli from human breast milk. 3 Biotech.

[269] Halloran K., Underwood M.A. (2019). Underwood, Probiotic mechanisms of action. Early Hum. Dev..

[270] Díaz-Ropero M., Martin R., Sierra S., Lara-Villoslada F., Rodríguez J.M., Xaus J., Olivares M. (2007). Two Lactobacillus strains, isolated from breast milk, differently modulate the immune response. J. Appl. Microbiol..

[271] Solís G., Reyes-Gavilan C.D.L., Fernández N., Margolles A., Gueimonde M., Reyes-Gavilán C.G.D.L. (2010). Establishment and development of lactic acid bacteria and bifidobacteria microbiota in breast-milk and the infant gut. Anaerobe.

[272] Rajoka M.S.R., Zhao H., Mehwish H.M., Li N., Lu Y., Lian Z., Shao D., Jin M., Li Q., Zhao L. (2019). Anti-tumor potential of cell free culture supernatant of Lactobacillus rhamnosus strains isolated from human breast milk. Food Res. Int..

[273] Gunyakti A., Özüsağlam M.A. (2019). Lactobacillus gasseri from human milk with probiotic potential and some technological properties. LWT.

[274] Mu Q., Tavella V.J., Luo X. (2018). Role of Lactobacillus reuteri in Human Health and Diseases. Front. Microbiol..

[275] Kosek M.N., Peñataro-Yori P., Paredes-Olortegui M., Lefante J., Ramal-Asayag C., Zamora-Babilonia M., Oberhelman R.A. (2019). Safety of Lactobacillus Reuteri DSM 17938 in Healthy Children 2–5 Years of Age. Pediatr. Infect. Disease J..

[276] Fatheree N.Y., Liu Y., Taylor C.M., Hoang T.K., Cai C., Rahbar M.H., Hessabi M., Ferris M., McMurtry V., Wong C. (2017). Lactobacillus reuteri for infants with colic: A double-blind, placebo-controlled, randomized clinical trial. J. Pediatr..

[277] Sung V., D’Amico F., Cabana M.D., Chau K., Koren G., Savino F., Szajewska H., Deshpande G., Dupont C., Indrio F. (2018). Lactobacillus reuteri to treat infant colic: A meta-analysis. Pediatrics.

[278] Rodenas C.L.G., Lepage M., Ngom-Bru C., Fotiou A., Papagaroufalis K., Berger B. (2016). Effect of formula containing Lactobacillus reuteri DSM 17938 on fecal microbiota of infants born by cesarean-section. J. Pediatr. Gastroenterol. Nutr..

[279] Francavilla R., Lionetti E., Castellaneta S., Ciruzzi F., Indrio F., Masciale A., Fontana F., La Rosa M.M., Cavallo L., Francavilla A. (2012). Randomised clinical trial: L actobacillus reuteri DSM 17938 vs. placebo in children with acute diarrhoea-a double-blind study. Aliment. Pharmacol. Ther..

[280] Dinleyici E.C., Dalgic N., Guven S., Metin O., Yasa O., Kurugol Z., Turel O., Tanir G., SamiYazar A., Sancar M. (2015). Lactobacillus reuteri DSM 17938 shortens acute infectious diarrhea in a pediatric outpatient setting. Jornal Pediatria.

[281] Dinleyici E.C., Vandenplas Y., PROBAGE Study Group (2014). Lactobacillus reuteri DSM 17938 effectively reduces the duration of acute diarrhoea in hospitalised children. Acta Paediatr..

[282] Urbanska M., Gieruszczak-Białek D., Szajewska H. (2016). Systematic review with meta-analysis: Lactobacillus reuteri DSM 17938 for diarrhoeal diseases in children. Aliment. Pharmacol. Ther..

[283] Gutiérrez-Castrellón P., López-Velázquez G., Garcia M.L.D., Jimenez-Gutierrez C., Mancilla-Ramirez J., Estevez-Jimenez J., Parra M. (2014). Diarrhea in preschool children and Lactobacillus reuteri: A randomized controlled trial. Pediatrics.

[284] Khalkhali S., Mojgani N. (2018). In vitro and in vivo safety analysis of Enterococcus faecium 2C isolated from human breast milk. Microb. Pathog..

[285] Bagci U., Togay S.O., Temiz A., Ay M. (2019). Probiotic characteristics of bacteriocin-producing Enterococcus faecium strains isolated from human milk and colostrum. Folia Microbiol..

[286] Fouhy F., Deane J., Rea M.C., O’Sullivan O., Ross P., O’Callaghan G., Plant B.J., Stanton C. (2015). The effects of freezing on faecal microbiota as determined using MiSeq sequencing and culture-based investigations. PLoS ONE.

[287] Lackey K.A., Williams J.E., Price W.J., Carrothers J.M., Brooker S.L., Shafii B., McGuire M.A., McGuire M. (2017). Comparison of commercially-available preservatives for maintaining the integrity of bacterial DNA in human milk. J. Microbiol. Methods.

[288] Fouhy F., Clooney A.G., Stanton C., Claesson M.J., Cotter P. (2016). 16S rRNA gene sequencing of mock microbial populations-impact of DNA extraction method, primer choice and sequencing platform. BMC Microbiol..

[289] Martín R., Bermúdez-Humarán L.G., Langella P. (2018). Searching for the bacterial effector: The example of the multi-skilled commensal bacterium Faecalibacterium prausnitzii. Front. Microbiol..

[290] Cani P.D., De Vos W.M. (2017). Next-generation beneficial microbes: the case of Akkermansia muciniphila. Front. Microbiol..

